# Catchment area and cancer population health research through a novel population-based statewide database: a scoping review

**DOI:** 10.1093/jncics/pkae066

**Published:** 2024-08-16

**Authors:** Lisa P Spees, Natasha Albaneze, Christopher D Baggett, Laura Green, Katie Johnson, Hayley N Morris, Ana I Salas, Andrew Olshan, Stephanie B Wheeler

**Affiliations:** Lineberger Comprehensive Cancer Center, University of North Carolina, Chapel Hill, NC, USA; Department of Health Policy and Management, Gillings School of Global Public Health, University of North Carolina, Chapel Hill, NC, USA; Lineberger Comprehensive Cancer Center, University of North Carolina, Chapel Hill, NC, USA; Department of Epidemiology, Gillings School of Global Public Health, University of North Carolina, Chapel Hill, NC, USA; Lineberger Comprehensive Cancer Center, University of North Carolina, Chapel Hill, NC, USA; Department of Epidemiology, Gillings School of Global Public Health, University of North Carolina, Chapel Hill, NC, USA; Lineberger Comprehensive Cancer Center, University of North Carolina, Chapel Hill, NC, USA; Lineberger Comprehensive Cancer Center, University of North Carolina, Chapel Hill, NC, USA; Lineberger Comprehensive Cancer Center, University of North Carolina, Chapel Hill, NC, USA; Lineberger Comprehensive Cancer Center, University of North Carolina, Chapel Hill, NC, USA; Lineberger Comprehensive Cancer Center, University of North Carolina, Chapel Hill, NC, USA; Department of Epidemiology, Gillings School of Global Public Health, University of North Carolina, Chapel Hill, NC, USA; Lineberger Comprehensive Cancer Center, University of North Carolina, Chapel Hill, NC, USA; Department of Health Policy and Management, Gillings School of Global Public Health, University of North Carolina, Chapel Hill, NC, USA

## Abstract

**Background:**

Population-based linked datasets are vital to generate catchment area and population health research. The novel Cancer Information and Population Health Resource (CIPHR) links statewide cancer registry data, public and private insurance claims, and provider- and area-level data, representing more than 80% of North Carolina’s large, diverse population of individuals diagnosed with cancer. This scoping review of articles that used CIPHR data characterizes the breadth of research generated and identifies further opportunities for population-based health research.

**Methods:**

Articles published between January 2012 and August 2023 were categorized by cancer site and outcomes examined across the care continuum. Statistically significant associations between patient-, provider-, system-, and policy-level factors and outcomes were summarized.

**Results:**

Among 51 articles, 42 reported results across 23 unique cancer sites and 13 aggregated across multiple sites. The most common outcomes examined were treatment initiation and/or adherence (n = 14), mortality or survival (n = 9), and health-care resource utilization (n = 9). Few articles focused on cancer recurrence (n = 1) or distance to care (n = 1) as outcomes. Many articles discussed racial, ethnic, geographic, and socioeconomic inequities in care.

**Conclusions:**

These findings demonstrate the value of robust, longitudinal, linked, population-based databases to facilitate catchment area and population health research aimed at elucidating cancer risk factors, outcomes, care delivery trends, and inequities that warrant intervention and policy attention. Lessons learned from years of analytics using CIPHR highlight opportunities to explore less frequently studied cancers and outcomes, motivate equity-focused interventions, and inform development of similar resources.

Prospective data linkage is a powerful tool for population health research, enabling the pairing of data from diverse sources to facilitate better understanding of associations between factors such as health outcomes, risk behaviors, health resource access and utilization, and demographic variables. Combining multilevel data from several sources enables the study of a greater number and a wider range of relationships compared with what is possible with each dataset in isolation. In particular, linking cancer registry data with other datasets, such as electronic health records, public and private administrative claims, vital statistics, census data, and area resource files, can facilitate the surveillance and study of trends and associations across the cancer care continuum. These linkages facilitate the conduct of population-based observational studies that can inform our understanding of cancer care and outcomes from a variety of perspectives and possible opportunities for intervention (1,2). These studies can help cancer centers better serve those in their catchment area, which is defined by the center and often represents the geographic area where patients and/or research participants live and/or where the cancer center markets itself (3). These studies also are opportunities for resource sharing and collaboration between cancer centers with overlapping catchment areas (4).

Many state cancer registries allow ad hoc linkage of their data to other health and demographic data, such as California, Texas, and Utah (5-10). For example, using linkages to medical records and area-level demographic data, a study of California Cancer Registry data examined associations between English proficiency and health-care engagement among breast cancer patients and another examined living in food deserts and breast and colorectal cancer mortality (5,6). Similarly, studies linking Texas Cancer Registry data with area-level demographic data, birth certificate data, and claims data examined associations between area-level socioeconomic status (SES) and cancer stage at diagnosis among patients with any type of cancer, demographic and perinatal factors among those with pediatric neuroblastoma, and patterns of care in pancreatic cancer patients (7-9). The Utah Cancer Registry was linked to the Utah Population Health Database to understand factors related to hereditary breast and ovarian cancer testing eligibility (10).

Although these studies have led to important population-level findings, they were singular studies that included 1-time linkages. In contrast, the National Cancer Institute (NCI)–designated Lineberger Comprehensive Cancer Center at the University of North Carolina (UNC) at Chapel Hill has developed the Cancer Information and Population Health Resource (CIPHR), through which North Carolina Central Cancer Registry (NCCCR) data are routinely linked with Medicaid, Medicare, and private payer claims data (2). As a permanent, continually updated resource, CIPHR provides researchers with access to a population-based cancer surveillance database that also includes patient information on real-world health-care resource utilization, patterns of treatment, costs of care, and health outcomes. In addition, CIPHR data have been linked to provider-level and health-care resources, area-level demographics, electronic health records, and environmental data.

The depth and breadth of studies conducted and published using CIPHR data have not been documented. Conducting a scoping review of such a rich resource of real-world data can help elucidate gaps in knowledge, which can be filled with future research, and areas where intervention is most needed, based on a high presence of risk factors or poor outcomes. In the following article, we review studies published using CIPHR data and key findings. We first mapped these articles onto the cancer care continuum (on the basis of study outcomes) and examined the frequency with which different types of cancer were included. Furthermore, using a multilevel socioecological framework, we reviewed key associations with health-related outcomes. These results were then summarized to elucidate what CIPHR has contributed to our understanding of population-level cancer prevention and control across the care continuum. Finally, we identified gaps in and future directions for population-level cancer research that can be addressed with linked data resources such as CIPHR. This information can inform the development of similar linked database resources in other geographic regions.

## Methods

### CIPHR overview

CIPHR, formerly known as the Integrated Cancer Information and Surveillance System (2), provides a prospective data linkage between NCCCR and statewide multipayer Medicare, Medicaid, and private insurance claims (11). For the present analysis, we included all articles published between 2012 and 2023 (which included cancer cases diagnosed between 2003 and 2020). The data cover more than 80% of individuals diagnosed with cancer in North Carolina and include claims data for cancer and noncancer patients, covering approximately 55% of the total North Carolina population. Additionally, CIPHR data can be linked to other available individual- and area-level datasets such as electronic medical records, birth certificates, North Carolina Medical Licensure data, Area Health Resource Files, and the Robert Wood Johnson County Health Rankings.

In addition to CIPHR’s robust, evolving data system, the resource program includes a team of data programmers, statisticians, health services researchers, and epidemiologists, who specialize in its use. CIPHR staff are also familiar with and support analyses using national datasets including Surveillance, Epidemiology, and End Results Program (SEER)–Medicare and MarketScan.

The CIPHR data are governed by agreements with several data partners and are not publicly available. However, in accordance with partner-specific data use agreements and approval processes, these data are available to researchers affiliated with the UNC. Additionally, researchers outside of UNC may access and use the CIPHR data by collaborating and coleading projects with a UNC-based partner. Our data use agreements ensure the scientific independence of any results.

### Search criteria

We used the Preferred Reporting Items for Systematic Reviews and Meta-Analyses for Scoping Reviews framework to guide this scoping review. To identify articles fitting our search criteria, we reviewed articles from CIPHR’s internal study database, which comprehensively documents all articles of studies with approved data use agreements and with analyses conducted by CIPHR analysts, published between January 2012 and August 2023 (12). Using this complete list, articles were reviewed independently by 2 reviewers (KJ and NA), and in the case of discrepancies, a third reviewer (LPS) was consulted until a consensus was reached. To be included, articles must have used CIPHR data (ie, state registry data linked to Medicare, Medicaid, and/or private insurance claims). Articles with analyses conducted by a CIPHR analyst but not including CIPHR data (such as those using SEER-Medicare or MarketScan data) were excluded.

**Figure 1.**
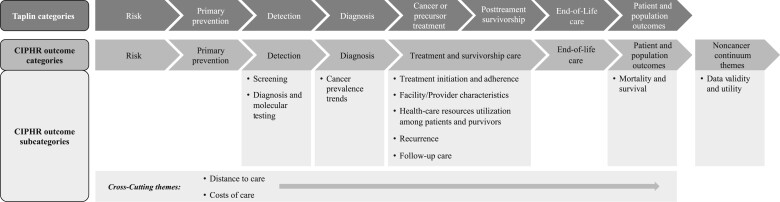
Outcomes across the cancer care continuum [based on Taplin et al. (13)]. To account for the American Cancer Society definition of *cancer survivorship*, which defines cancer survivor as any individual diagnosed with cancer, we combined the categories “cancer or precursor treatment” and “post-treatment survivorship” into a single category, “treatment and survivorship care.” Within this category, we divided articles into outcome subcategories based on themes such as “treatment initiation and adherence,” “treatment site/provider characteristics,” “recurrence,” “follow-up care,” and “health care resource utilization among patients and survivors.” We also included 2 categories that spanned across the cancer care continuum: “costs of care” and “distance to care.” CIPHR = Cancer Information and Population Health Resource.

Articles were abstracted independently by KJ and NA using structured fields, specifically title, author, major topics of study, types of cancer(s), sample size, whether results were stratified by race and ethnicity and/or rurality, key results, and payer type (Medicare, Medicaid, and/or private) (Supplementary Table 1, available online). Discrepancies were again resolved by consulting LPS and aligning on a consensus. Articles were categorized based on the article’s main outcomes in relation to where the outcomes fell across the Taplin et al. (13) cancer care continuum and then bucketed into more specific outcome subcategories. Articles with outcomes or analyses that fell outside of the scope of the cancer care continuum (eg, data validity and utility) were added as additional categories. (See Figure 1 for a comprehensive list of cancer care continuum categories and subcategories.) The number of articles within each outcome subcategory and across each cancer site were calculated, and the cross-tabulation of theme and cancer site were quantified to elucidate what topics have been frequently studied and where gaps remain. Finally, we wanted to summarize the analyses and results across the articles. Thus, for each article, notable results and/or conclusions were examined, particularly statistically significant associations with patient-level factors, including sociodemographics, as well as treatment- and access-related factors (eg, insurance type, distance to care). We also examined associations with provider- and facility-level factors. For articles that focused on 1 specific relationship, as opposed to myriad potential associations, we noted whether the relationship was statistically significant. Results of articles evaluating the same outcomes in patients with the same cancer type were compared, when appropriate.

## Results

As of September 2023, a total of 130 articles published between January 2012 and August 2023 were initially identified for review. During the review process, 79 articles were removed because they did not include CIPHR data. In total, 51 articles were identified for inclusion; 43 of the 51 articles reported results for 1 or more individual cancer sites, and 13 of the 51 articles reported results aggregated across multiple cancer sites.

**Figure 2.**
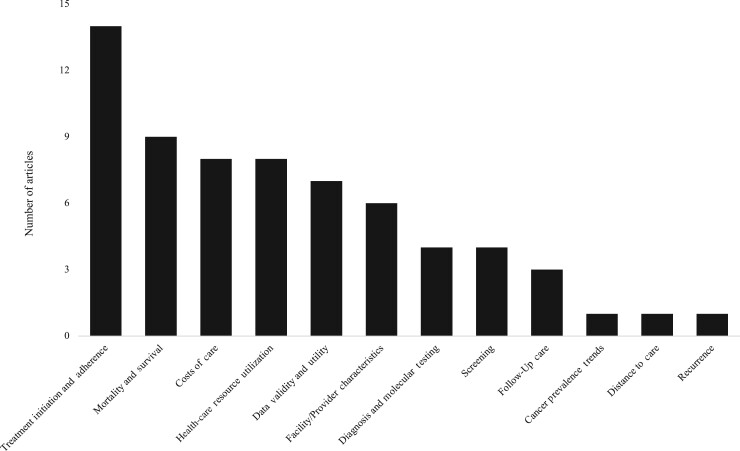
Cancer Information and Population Health Resource articles, stratified by cancer care continuum outcome subcategory.

Across the cancer care continuum, the majority of articles were categorized as part of treatment and survivorship care (n = 27) (Table 1); within this category, most articles examined treatment initiation and/or adherence outcomes (n = 14) (Figure 2). In several articles, distance to care and insurance status and/or type at diagnosis—key variables that can be measured using the CIPHR data—were found to be statistically significantly associated with treatment-related outcomes (14-20).

**Table 1.** Cancer Information and Population Health Resource articles across the cancer care continuum

Cancer care continuum outcomes	Outcome subcategory	Cancer Information and Population Health Resource articles (reference numbers)	Cancer types included
Detection	Screening	(39-41,49)	Colorectal, liver
	Diagnosis and molecular testing	(21,46,47,50)	Breast, cervical, ovarian, prostate, uterine, vaginal, multisite aggregated
Diagnosis	Cancer prevalence trends	(22)	Breast
Treatment and survivorship care	Treatment initiation and adherence	(14,16-20,23-28,42,51)	Breast, cervical, colorectal, kidney, lung, melanoma
	Facility and provider characteristics	(20,29,42,48,52,53)	Acute myeloid leukemia, colorectal, multiple myeloma, uterine, multisite aggregated
	Health-care resource utilization among cancer patients and survivors	(30,31,54-59)	Bladder, breast, colorectal, lung, melanoma, prostate, multisite aggregated
	Recurrence	(25)	Breast
	Follow-up care	(15,32,42)	Aggregated multisite
Patient and population outcomes	Mortality and survival	(42,46-48,52,53,59-61)	Acute myeloid leukemia, bladder, cervical, colorectal, kidney, melanoma, ovarian, uterine, vaginal, multisite aggregated
Cross-cutting themes	Distance to care	(29)	Breast, colorectal, esophageal, gallbladder, liver, lung, melanoma, pancreatic, stomach
	Costs of care	(22,31,33-36,43,58)	Breast, colorectal, head and neck, lung, multisite aggregated
Noncancer care continuum themes	Data validity and utility	(37,38,44,45,62-64)	Breast, cervical, colorectal, Hodgkin lymphoma, non-Hodgkin lymphoma, ovarian, uterine, multisite aggregated

Other common outcomes included mortality and survival (n = 9) and health-care resource utilization among patients and survivors (n = 9) (Figure 2). In contrast, there was a dearth of literature examining associations with cancer recurrence (n = 1), distance to cancer care (n = 1), or cancer prevalence trends (n = 1) (Figure 2).

**Figure 3.**
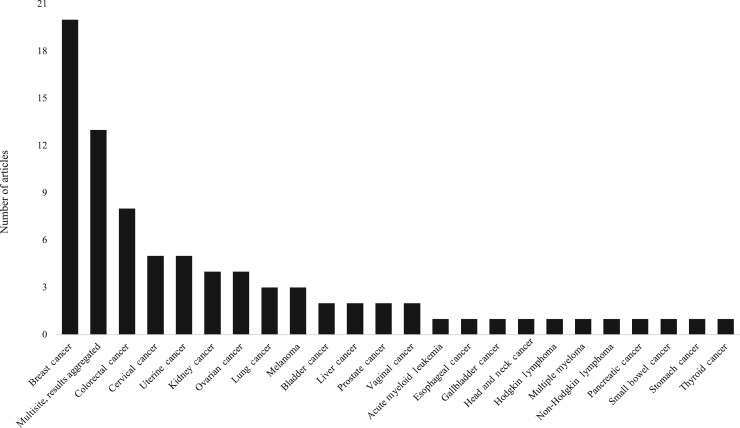
Cancer Information and Population Health Resource articles by cancer type.

**Figure 4.**
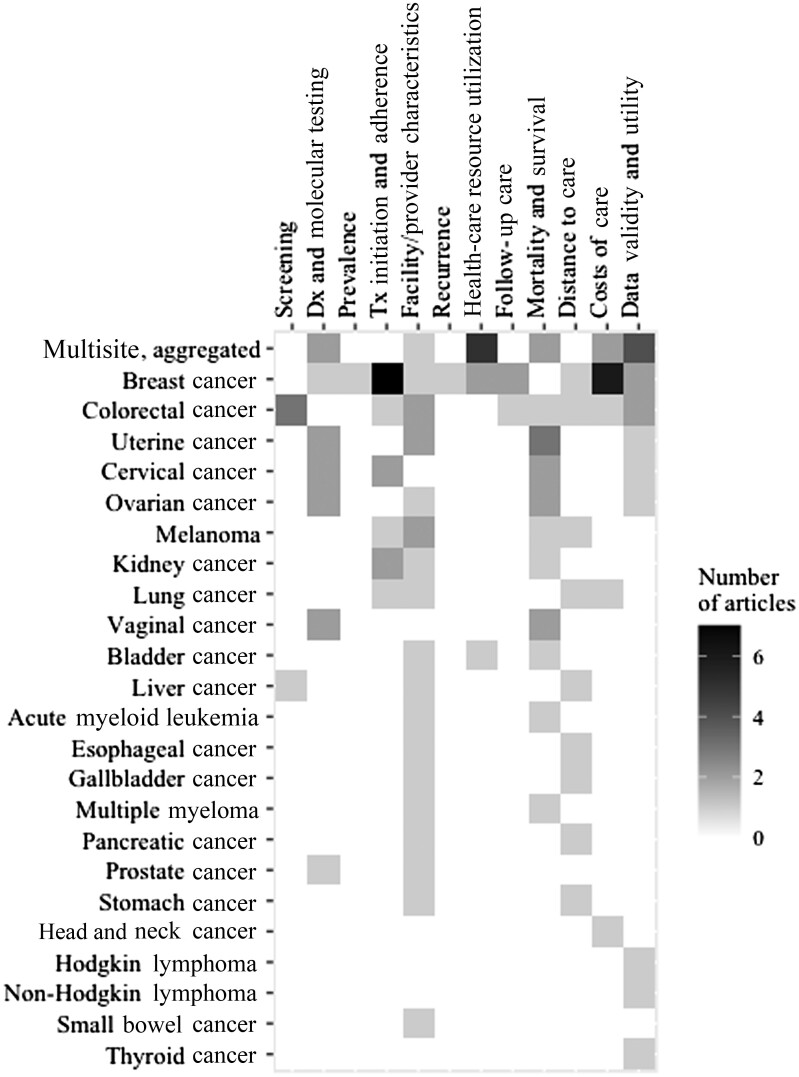
Cross-tabulation of Cancer Information and Population Health Resource articles, by cancer site and cancer care continuum outcome subcategory. Articles may appear more than once when more than 1 cancer site and/or outcome subcategory was examined. Dx = diagnosis; Tx = treatment.

Of the 23 individual (ie, nonaggregated) cancer sites examined by the CIPHR articles, the site most frequently examined was breast cancer (Figure 3), which was analyzed in 20 articles (15,16,21-38) across 10 cancer care continuum categories (Figure 4). Articles on breast cancer examined a range of outcomes, from receipt of molecular testing (21) to treatment (16,23-28) to follow-up care (15,32). Notably, articles focusing on prevalence (22) and recurrence (25) outcomes were conducted solely among individuals with breast cancer.

After breast cancer, the next most frequently examined individual cancer sites included colorectal (n = 8) (29,39-45), cervical (n = 5) (17,18,37,46,47), and uterine cancer (n = 5) (29,37,46-48). Eleven cancer sites were examined in only 1 article each.

Next, we describe the CIPHR articles across key outcome subcategories.

### Screening

Four articles focused on cancer screening across 2 cancer sites (Table 1) (39-41,49).

Two articles examined factors associated with receipt of colorectal cancer screening (39,40). Per US Preventive Services Taskforce guidelines, three-quarters of people aged 59-75 years were up-to-date on screening (39). One article found that those with any private insurance were more likely than those with only Medicare to be up-to-date on screening (39). Generally, women were more likely than men to receive and be up-to-date on screening (39,40). In addition, those who lived farther from a screening facility were less likely to receive and be up-to-date with colorectal cancer screening (39,40).

A third colorectal cancer–focused article examined type of testing received—colonoscopy vs fecal occult blood test or fecal immunochemical test—among publicly and privately insured individuals (41). Privately insured individuals were more likely to be screened via fecal occult blood test or fecal immunochemical test (58%), whereas publicly insured individuals were more likely to be screened via colonoscopy (57%). Among all individuals who were screened, women were less likely to receive colonoscopy over fecal occult blood test or fecal immunochemical test compared with men.

One article focused on the incidence of hepatocellular carcinoma screening, stratified by insurance type and cirrhosis etiology (49). Approximately one-half of cirrhosis patients were screened for hepatocellular carcinoma screening in a given year between 2010 and 2016. However, after an initial drop in 2011, there was a yearly increase in the annual screening incidence that was driven primarily by an increase in screening among patients with Medicaid. In terms of etiology, patients with hepatitis B virus were most likely to be screened, followed closely by patients with hepatitis C virus, alcohol-related liver disease, and nonalcoholic fatty liver disease.

### Diagnosis and molecular testing

Four articles examined receipt of molecular testing or stage at diagnosis as outcomes across 6 cancer sites (Table 1) (21,46,47,50).

One article examined the association between race, provider characteristics, and likelihood of molecular testing in breast cancer patients (21). Patients treated by medical oncologists, as opposed to surgical or gynecologic oncologists, were more likely to receive gene expression profile testing. Lymph node–negative patients were more likely to receive gene expression profile testing if they had any private insurance at diagnosis or were treated by a physician with lower patient volumes and less likely to receive gene expression profile testing if they were non-Hispanic Black women. In contrast, lymph node–positive patients were less likely to receive gene expression profile testing if they were treated by physicians with lower patient volumes.

One article focused on the association between distance to care and the likelihood of high-risk prostate cancer (50). Greater distance to the nearest urologist was associated with an increased likelihood of high-risk prostate cancer, particularly for Black individuals.

Last, 2 articles focused on insurance coverage and stage at diagnosis among patients with gynecological (cervical, ovarian, uterine, vaginal) cancer (46,47). Those with Medicaid coverage in the 6 months before their cancer diagnosis were less likely to be diagnosed with advanced stage cancer than those with Medicaid coverage that started after their cancer diagnosis (47). Additionally, among uterine cancer patients aged 65 years and older, those with both Medicare and Medicaid were more likely to be diagnosed with late-stage disease than those with Medicare only (46).

### Cancer prevalence trends

One article forecasted future prevalence of metastatic breast cancer and predicted a 55% increase from 2015 to 2030, primarily driven by increased prevalence among women ages 18-44 years and 45-64 years (Table 1) (22).

### Treatment initiation and adherence

A total of 14 articles explored factors associated with receipt of appropriate treatment across 6 cancer sites (14,16-20,23-28,42,51). (Table 1).

Eight articles found myriad patient demographic and clinical factors were associated with likelihood of receipt of treatment (19,20,23-27,51). For example, higher burden of comorbidities and older age at diagnosis were associated with lower likelihood of treatment (eg, radiation therapy [RT], endocrine therapy) initiation among breast (23,25,27) and metastatic kidney cancer patients (19,51), with non-White race being an additional factor associated with lower likelihood of endocrine therapy initiation among breast cancer patients (24). Patient age was associated with type of surgical treatment for early breast cancer, with patients aged younger than 50 years more likely to undergo mastectomy and patients aged 50 years and older likely to undergo breast-conserving surgery (26). Among metastatic kidney cancer patients, those diagnosed with stage II disease were more likely than those diagnosed with stage IV disease to initiate oral anticancer agent treatment within 12 months of metastatic diagnosis (51). In melanoma patients, those with races or ethnicities other than non-Hispanic White or with cancer stage of II or III at diagnosis were more likely to have delayed time to surgery (20).

Insurance type was associated with treatment receipt in 2 articles (19,20). Among metastatic kidney cancer (19) and melanoma (20) patients, not having private insurance was associated with lower treatment adherence and delayed receipt of surgery, respectively.

The influence of distance to care was examined in 3 articles, often with different results by rurality of patient residence (16-18). Farther distance to care was often associated with lower likelihood of receipt of appropriate care among urban cervical cancer patients but increased likelihood of receipt of guideline concordant care among rural patients (17,18). For breast cancer patients, although urban residence was associated with increased likelihood of receipt of RT compared with rural residence, longer distance to care was associated with lower likelihood of RT among urban patients (16).

In 1 article on lung cancer patients, higher rates of any treatment were seen in those who had shorter distances to care (14). Although the likelihood of RT increased over time and the likelihood of surgical treatment has decreased, higher rates of surgical treatment were seen in those who lived closer to (vs farther from) a surgeon or in an area with low (vs high) radiation oncologist density.

Provider-level factors were examined in 4 articles (20,23,28,42). Melanoma patients were less likely to have delayed time to surgery if they were diagnosed by or if their surgeon was a dermatologist (20). In breast cancer patients, participating in the Breast and Cervical Cancer Control Program and visiting a medical oncologist within 1 year of cancer diagnosis each were associated with increased likelihood of endocrine therapy initiation (23,28). Colorectal cancer patients were more likely to receive adjuvant chemotherapy if surgeons and medical oncologists each shared more than approximately 10%-20% of their patient volume with the other specialty (42).

### Facility and provider characteristics

In total, 5 articles explored facility or provider characteristics as outcomes across 5 unique cancer sites, and 1 explored these outcomes across multiple aggregated cancer sites (Table 1) (20,29,42,48,52,53).

Three articles focused on provider specialty (20,29,42). For cancer patients who received surgery, those treated by general surgeons were more likely to be older, female, minoritized races or ethnicities, and from areas of high poverty compared with those treated by cancer site–specific surgical subspecialties (29). Among patients with melanoma, those who were older were more likely to be diagnosed by a dermatologist, while those who were publicly insured (eg, Medicaid or Medicare), were a race or ethnicity other than non-Hispanic White, lived in a rural zip code, had higher stage cancer, and had greater comorbidity burden were less likely to be diagnosed by a dermatologist than those with private insurance (20). Those diagnosed by a dermatologist were more likely to have surgery performed by a dermatologist, but those who had Medicaid (vs private insurance), lived in a rural county, and had higher stage cancer were less likely to have surgery performed by a dermatologist (20). Among colorectal cancer patients, those whose surgeon shared more than 40% (vs 40% or less) of their patient volume with a medical oncologist were more likely to have a consultation with a medical oncologist; however, patients whose surgeon had greater colorectal cancer patient volume were less likely to have a consultation with a medical oncologist (42).

Two articles focused on facility characteristics (52,53). In multiple myeloma patients, younger age, private insurance, a Charlson comorbidity index score of at least 3.0, a higher activity of daily living dependency score, and a longer distance to the nearest NCI-designated comprehensive cancer center facility were associated with lower likelihood of treatment at a NCI-designated comprehensive cancer center (52). In acute myeloid leukemia patients, farther distance (vs nearest) to an actual treatment facility was associated with increased likelihood of treatment at an NCI-designated comprehensive cancer center (vs non–NCI-designated comprehensive cancer center treatment facility) (53).

One article focused on referral patterns of uterine cancer patients and facility volume (48). In this article, those who received their treatment at low-volume centers were more likely to be older and publicly insured, live in nonmetro counties, and have higher comorbidity scores.

Those who received their initial biopsy at a low-volume facility were more likely to be referred to a high-volume facility for treatment if they were diagnosed with a high-risk histology but less likely if they had Medicaid (vs private insurance).

### Health-care resource utilization among cancer patients and survivors

Eight articles analyzed factors associated with health-care resource utilization among cancer patients and survivors, with 3 articles focused on 2 individual cancer sites and 5 articles across multiple aggregated cancer sites (Table 1) (30,31,54-59).

Two articles focused on use of opioids and/or benzodiazepines (54,55). Overall, approximately 15% of cancer patients were coprescribed opioids and benzodiazepines, with the highest rates seen among cervical (31%) and ovarian (26%) cancer patients (55). Younger patients, patients with regional (vs local) disease, and patients with comorbidities were more likely to be coprescribed opioids and benzodiazepines (55). Among cancer survivors, factors associated with increased likelihood of chronic opioid use included prior opioid use, prior depression, chronic pain, or substance use disorder diagnoses, and higher comorbidity burden (54).

One article examined use of occupational therapy (56). Among breast, colorectal, lung, prostate cancer, and melanoma patients, demographic factors such as older age, female sex, greater educational attainment, history of occupational therapy use, diagnosis at stage I-III, and greater comorbidity burden were positively associated with use of occupational therapy postdiagnosis.

One article explored the relationship between hospital readmissions and distance to cystectomy provider and found no association with either 30- or 90-day readmission (59).

Four articles focused on inpatient hospitalizations and emergency department (ED) visits (30,31,57,58). Among patients with cardiometabolic conditions, cancer patients had a higher likelihood of hospitalization compared with matched noncancer patients (58). Among survivors aged 65 years or older of any cancer, increased frailty and impairments in activities of daily living were associated with an increased likelihood of hospitalization and long-term care visits (57).

A subset of these articles specifically examined patient-centered medical home enrollment (30,31,58). One article examining breast cancer patients reported patient-centered medical home enrollment was not associated with hospitalizations or ED visits (31). In another article focused on breast, colorectal, and lung cancer patients with cardiometabolic conditions, there was no difference in ED visits between those enrolled in patient-centered medical home vs those who are not (58). However, there was an increase in hospitalizations seen in cancer relative to noncancer patients, and in the hypertension subgroup, the increase was larger among those enrolled in a patient-centered medical home than those not enrolled in a patient-centered medical home. Another article found that patient-centered medical home enrollment was associated with a decreased likelihood of inpatient admission for chemotherapy-related adverse events in breast cancer patients (30). Regarding outpatient care, patient-centered medical home enrollment was associated with an increased likelihood of outpatient visits unrelated to breast cancer treatment (31).

### Recurrence

One article examined factors associated with recurrence among breast cancer patients (Table 1) (25). Older age and an increased burden of comorbidities were found to be associated with an increased likelihood of recurrence. Prior treatment with RT with or without endocrine therapy, as opposed to endocrine therapy only, was associated with a lower likelihood of recurrence.

### Follow-Up care

Three articles examined follow-up care among survivors across 2 individual cancer sites (Table 1) (15,32,42).

In 1 article among breast cancer patients, non-White race, having Medicare or Medicaid insurance, increased distance to a surgeon, living in a rural county, and having more advanced disease were associated with lower likelihood of postmastectomy breast reconstruction (15).

Among breast cancer survivors, increased duration of patient-centered medical home enrollment was associated with greater likelihood of surveillance mammography (32). Colorectal cancer survivors who underwent surgery were more likely to receive surveillance colonoscopy if their surgeon had a large colorectal cancer patient volume or if their surgeon and medical oncologist each shared more than approximately 10%-20% of their patient volume with the other specialty (42).

### Mortality and survival

Factors associated with mortality or survival were analyzed in 9 articles across 10 cancer sites (Table 1) (42,46-48,52,53,59-61).

Four articles examined patient and treatment factors associated with mortality (53,59-61). Among metastatic kidney cancer patients, having a de novo (vs recurrent) metastatic diagnosis and having increased frailty were associated with increased risk for mortality (61). In acute myeloid leukemia patients, older age, having a higher burden of comorbidities, and not receiving inpatient chemotherapy or timely allogeneic hematopoietic stem cell transplantation were associated with higher risk for 1-year mortality (53). Similarly, delayed surgical excision for melanoma was associated with lower survival up to 5 years postsurgery (60). In bladder cancer patients who underwent cystectomy, those who were readmitted within 90 days of discharge, experienced a major complication postcystectomy, had more advanced pathological stage, or had increased comorbidity burden had lower long-term survival (59).

Three articles examined the association between insurance coverage and mortality (46,47,61). Among metastatic kidney cancer patients, having Medicare and no private insurance was associated with increased risk for mortality (61). Gynecologic cancer patients aged 65 years and older with only Medicare coverage had lower likelihood of mortality than those with dual enrollment (ie, Medicare and Medicaid) (46). Another article showed no difference in mortality between gynecologic cancer patients with prediagnosis Medicaid coverage and those covered postdiagnosis (47).

Three articles analyzed the relationship between provider or facility characteristics and survival (42,48,52). Receiving treatment at an NCI-designated comprehensive cancer center (compared with a non–NCI-designated comprehensive cancer center) or high-volume uterine center (as opposed to a low-volume center) was associated with lower risk for mortality among multiple myeloma and uterine cancer patients, respectively (48,52). Lastly, neither individual nor shared colorectal cancer patient volumes of surgeons and medical oncologists were associated with 5-year overall survival for colorectal cancer patients (42).

### Distance to care

One article examined distance to care as an outcome in 15 cancer sites (Table 1) (29). Among each of bladder, breast, colorectal, esophageal, gallbladder, kidney, liver, lung, melanoma, ovarian, pancreatic, prostate, small bowel, stomach, and uterine cancer patients receiving surgical treatment, distance to actual treatment facility was farther for patients seeing specialists than those seeing general surgeons.

### Costs of care

Seven articles examined cancer care costs across 4 cancer sites, and 1 did so across multiple aggregated sites (Table 1) (22,31,33-36,43,58).

Overall health-care costs were higher among breast cancer patients than noncancer patients (33-35) and increased as cancer stage increased (34,35). However, breast cancer treatment type (ie, RT vs endocrine therapy vs both) was not associated with cost (or quality-associated life-years) (36).

Medical and productivity costs associated with metastatic breast cancer were forecasted to increase to 140% of 2015 levels by 2023 (22). The plurality of the medical and productivity cost burdens was predicted to mostly affect women aged 45-65 years and 18-44 years, respectively (22).

The association between facility characteristics and costs varied across articles (31,43,58). Among breast, colorectal, and lung cancer patients with chronic metabolic conditions, cancer patients had statistically significantly increased expenditures relative to noncancer patients, but those enrolled in a patient-centered medical home had smaller increases than patients not enrolled in a patient-centered medical home (58). However, in another article, being enrolled in a patient-centered medical home was associated with higher monthly expenditures in the first 15 months postbreast cancer diagnosis (31). In colorectal, head and neck, and lung cancer patients, no statistically significant difference was found between NCI-designated comprehensive cancer centers and non–NCI-designated comprehensive cancer centers in terms of use of high-cost treatment options overall, but among the privately insured subgroup of patients, those not treated at NCI-designated comprehensive cancer centers were more likely to receive high-cost treatments than those treated at NCI-designated comprehensive cancer centers (43).

### Data validity and utility

Six articles focused on cancer registry data validity and utility: 4 across 8 individual cancers and 3 across multiple aggregated sites (Table 1) (37,38,44,45,62-64).

Two articles examined the validity of treatment data in the cancer registry relative to insurance claims (37,38). The sensitivity and positive predictive value of cancer registry data on receipt of treatment relative to claims data varied by treatment and cancer type but were generally more than 65% (37,38). However, there was variation by insurance type and the strength, and direction of associations were not consistent across articles (37,38). The degree to which the timing of treatment initiation matched between registry and claims data also varied based on the treatment received (37,38). Notably, 1 of the articles reported that breast cancer patients with linked registry and claims data were more likely than those whose data was not linked to be Black patients and living in rural counties (38).

Two articles evaluated the potential for linking cancer registry data to other data sources, such as health system data, claims data, other cancer registries, vital records, and survey data (62,63). Generally, the researchers saw potential in these linkages for passive data collection and follow-up to limit potential participant burden, attrition, and selection biases (62,63).

One article described the feasibility of a colorectal cancer provider network analysis using claims data (45). Patients with private insurance (vs Medicare) were more likely to be shared by providers of 2 or more specialties, and providers in the Medicare (vs private) network shared patients with a greater total number of other providers (45). Across payers, provider location (within the patient volume distribution), clustering, betweenness, and centrality differed (45).

In 1 article, the NCCCR was determined to be an effective NCI clinical trial enrollment surveillance tool (64). The trial enrollment rate was higher for White cancer patients than minoritized cancer patients overall and when stratified by sex (64). Additionally, greater enrollment in a given county was associated with presence of a medical school or NCI community clinical oncology program–affiliated practice in that county.

Lastly, 1 article evaluated 4 SES measures to determine which best improved model predictions of receipt of guideline-concordant care and overall survival in colorectal cancer patients (44). Three measures, the Social Deprivation Index, the Social Vulnerability Index, and the Area Deprivation Index, improved model prediction of receipt of guideline-concordant colorectal cancer care, whereas the fourth SES measure, the Distressed Community Index, did not. However, models with each of the 4 SES measures had the same area under the curve. In terms of predicting overall survival in colorectal cancer patients, all 4 SES measures improved model prediction, and models with each measure had the same area under the curve. Thus, SES measures are important to include in models predicting colorectal cancer patient outcomes, but no individual measure stands out as superior.

## Discussion

In this review, we included 51 articles that used a novel data resource, CIPHR, which links registry data to multipayer claims data. Articles examined myriad topics including 1) trends in treatment and survivorship care, 2) variation in costs, health-care utilization, and cancer outcomes among marginalized populations (eg, survival disparities in racial and ethnic minoritized populations, rural and low-income populations), and 3) multilevel predictors (eg, patient, provider, and health system factors) of cancer outcomes. By maintaining and regularly updating this unique data linkage, CIPHR enables researchers to examine a wide breadth of study topics.

The findings of this review illustrate existing literature gaps and highlight areas where future cancer-related health services research is needed. For example, among our 12 cancer care continuum categories, only 2 (cancer treatment facility and provider characteristics and mortality and survival) were examined in the context of at least 10 unique cancer sites (of 23 total). Additionally, only 2 unique cancer sites, breast and colorectal cancer, were examined in the context of more than 5 different outcomes, and most were only examined in the context of 1 or 2 outcomes. Even when there were multiple articles examining the same outcome, there was often a focus on only a few cancers. For example, despite being among the most common outcomes of interest, treatment initiation and/or adherence were examined only in the context of 5 unique cancer sites.

The high incidence of breast cancer, as well as the presence of racial disparities in breast cancer incidence, treatment, and mortality outcomes, has contributed to breast cancer being the most studied cancer in the United States and using CIPHR resource data. However, there is a dearth of literature using CIPHR for other commonly diagnosed cancers, such as prostate and lung cancer, both of which have incidence and mortality rates that are higher in North Carolina than the United States as a whole (65). Additionally, despite the known racial and geographic disparities among patients with cancer types such as stomach and myeloma, no CIPHR studies have examined the factors contributing to these disparities. Further research is warranted to better understand the drivers and determinants of location-specific cancer incidence and mortality trends.

Regarding gaps in specific cancer care continuum outcomes, this scoping review illustrates that more research is needed beyond cancer mortality and treatment initiation. A notable gap in the literature relates to the establishment and evaluation of reliable quality care indicators across treatment, survivorship, and end-of-life care. Keating et al. (66) used SEER registry and Medicare claims data to assess the reliability of various cancer treatment, surveillance, survival, and end-of-life care quality measures. They found the quality measures, which were adapted from those recommended by groups such as the National Quality Forum, the American College of Surgeons, the American Society of Clinical Oncology, and the National Comprehensive Cancer Network, had limited reliability, pointing to the need for development of quality care measures that can be consistently and accurately identified in claims data (66). Earle et al. (67) identified end-of-life care quality indicators that can be measured in administrative data, such as claims; however, they did not assess the reliability of these measures. The articles in this review illustrate the potential that CIPHR may have in developing, evaluating, and refining a set of meaningful and reliable measures to be used for evaluating care quality.

Two additional gaps in the current CIPHR literature should be noted. First, there is a lack of focus on pediatric and adolescent cancers. None of the articles focused specifically on pediatric cancer patients. Two articles related to data validity and utility focused on data in adolescent and young adult populations; the findings of both support the utility of CIPHR for the study of certain outcomes in adolescent and young adult cancer patient populations (37,62). Second, the availability of claims data through CIPHR, regardless of patient cancer status, allows for the study of cancer screening trends and associated factors using CIPHR. Thus, with only 4 screening-focused articles across 2 cancer sites within the CIPHR literature set, screening-focused articles represent a surprising gap.

This scoping review has a few limitations that should be noted. First, projects and studies that used CIPHR, but resulted in abstracts or conference presentations without a fully published article, were not included. This may have resulted in incomplete reporting of the types of outcomes that have been studied using CIPHR data. Second, the generalizability of our findings may be limited to a single state and may not fully represent the overall trajectory of linked data literature across the cancer care continuum. However, because North Carolina is a large state, with a racially, geographically, and socioeconomically diverse population, our findings still provide initial guidance on where future research is needed.

This scoping review has illustrated the value of the CIPHR database, and indeed registry-linked-claims data more generally, as a population-level cancer research and care monitoring resource, enabling the study of diverse topics across the cancer care continuum. The results of these studies can be used to identify priorities for intervention and policy generation. For example, CIPHR studies have been essential to identifying colorectal cancer hotspots in North Carolina (40). Based on these findings, colorectal cancer screening interventions were targeted in these hotspot counties (68). Other states or cancer centers that create CIPHR-like resources could work similarly with members of their catchment area to drive community engagement and impactful intervention.

The population-level, cancer care continuum–spanning analyses seen in the articles discussed in this review are made possible through the CIPHR database’s unique linkages of cancer registry, claims, provider, and area-level data. With major changes to public claims data access proposed recently by the Centers for Medicare and Medicaid Services, including the increased cost to use Medicare data, the ability to link Centers for Medicare and Medicaid Service data with state cancer registry and other data could be negatively affected starting in 2025 (69). Therefore, it is important for administrators and policy makers to understand the breadth and depth of research to improve patient outcomes made possible through linked data resources such as CIPHR—as described in this review.

While we demonstrate the potential of CIPHR to facilitate and provide impactful research, we also identify opportunities for future research. By summarizing the findings across studies using CIPHR data, we aim to generate more targeted research to fill the identified knowledge gaps in addition to initiating the development of interventions that build on current research findings. Although the CIPHR data is specific to North Carolina, findings from similar analyses in other population-based registry systems could help inform and prioritize local initiatives and policies. Thus, we hope these findings serve as a model for how investment by other states in a CIPHR-like resource could promote more widespread, impactful population-level cancer research.

## Supplementary Material

pkae066_Supplementary_Data

## Data Availability

No new data were generated or analyzed for this review.
