# Supplementary material for: Catchment area and cancer population health research through a novel population-based statewide database: a scoping review
Source: JNCI Cancer Spectr. 2024 Aug 16;8(5):pkae066. doi: 10.1093/jncics/pkae066 (PMC11410196; doi:10.1093/jncics/pkae066)
Supplement: pkae066_Supplementary_Data [file pkae066_supplementary_data.docx]

# Supplementary Table 1. Key Details of Included Articles

# Cancer Care Continuum Stage: Detection

## Theme: Screening

| Study | Topic(s) of Study  *(**within theme)* | Type of Screening | Sample Size | Stratified by/Adjusted for Race/Ethnicity?* | Stratified by/Adjusted for Rurality?* | Payer Type | Key Findings |
| --- | --- | --- | --- | --- | --- | --- | --- |
| Lich et al. 2023  PMID: 37285065 | - Association between several patient and regional demographic and access-related factors and being up to date with CRC screening (per USPSTF guidelines) | Colorectal | 274660 | Yes  *(quartiles of non-white resident proportion)* | No | Medicare, Private | Overall, from 2012-2016, 75% of the sample, aged 59-75, was up-to-date  Factors significantly with i**ncreased likelihood** being up to date on screening**:**   - Female gender (vs. male) - Age 60-64, 65-69, 70-72, or 73-75 at screening (vs. 59) - Screening in 2013, 2014, 2015, or 2016 (vs 2012) - Primary care visit within 12 months of screening - 1, 2, or 3+ comorbidities (vs. 0) - Any private insurance (vs. Medicare only) - 2^nd^, 3^rd^, or 4^th^ quartile of % residents who are non-White (vs, 1^st^) - Above median number of primary care physicians/10,000 persons (vs. below median)   Factors significantly with **decreased likelihood** of being up to date on screening**:**   - Living 5-10, 10-15, 15-20, 20-25, or ≥25 mi from nearest endoscopy facility (vs. 0-5 mi) - 2^nd^, 3^rd^, or 4^th^ quartile of % residents with less than a high school education (vs. 1^st^ quartile) - 2^nd^ or 4^th^ quartile of area unemployment rate (vs. 1^st^) - 2^nd^, 3^rd^, or 4^th^ quartile of % residents without insurance (vs. 1^st^ quartile) |
| Wheeler et al. 2016  PMID: 28210537 | - Association between several patient and regional demographic, access-related, and provider-related factors, and receipt of colonoscopy (vs. FOBT/FIT); stratified by payer type | Colorectal | 68721 | Yes, for publicly insured  *c* | No | Medicare, Medicaid, Private | Private: 42% colonoscopy / 58% FOBT/FIT  Public: 57% colonoscopy / 43% FOBT/FIT  Factors significantly associated with i**ncreased likelihood** of colonoscopy (vs. FOBT/FIT) among **privately insured:**   - Turning 50 in 2003, 2004, 2005, 2006, or 2007 (vs. 2008) - Ever enrolled in SEHP - 4^th^ regional % non-white population quartile (vs. 1^st^ quartile)   Factors significantly associated with d**ecreased likelihood** of colonoscopy (vs. FOBT/FIT) among **privately insured:**   - Female gender (vs. male) - Distance to nearest endoscopy facility 10-15 mi (vs. <5) - 2^nd^ regional unemployment rate quartile (vs. 1^st^ quartile)   Factors significantly associated with **increased likelihood** of colonoscopy (vs. FOBT/FIT) among **Medicare-only**:   - Distance to nearest endoscopy facility 10-15 or >20 mi (vs. <5) - Turning 50 in 2003, 2004, 2006, or 2007 (vs. 2008) - Endoscopy facility test volume 400-599 per 10,000 (vs. 0 per 10,000) - 3^rd^ or 4^th^ regional % non-white population quartile (vs. 1^st^ quartile)   Factors significantly associated with **decreased likelihood** of colonoscopy (vs. FOBT/FIT) among **Medicare-only**:   - Female gender (vs. male) - 3^rd^ or 4^th^ regional % unemployment quartile (vs. 1^st^ quartile)   Factors significantly associated with **increased likelihood** of colonoscopy (vs. FOBT/FIT) among **Medicaid-only**:   - Turning 50 in 2003, 2004, 2005, 2006, or 2007 (vs. 2008)   Factors significantly associated with **decreased likelihood** of colonoscopy (vs. FOBT/FIT) among **Medicaid-only**:   - Female gender (vs. male) - 3^rd^ regional uninsurance quartile (vs. 1^st^ quartile) - 3^rd^ regional unemployment quartile (vs. 1^st^ quartile)   Factors significantly associated with **increased likelihood** of colonoscopy (vs. FOBT/FIT) among **dual-enrolled:**   - Turning 50 in 2003 (vs. 2008)   Factors significantly associated with **decreased likelihood** of colonoscopy (vs. FOBT/FIT) among **dual-enrolled**:   - Female gender (vs. male) - Other (non-white and non-Black) race (vs. white) |
| Wheeler et al. 2014 (a)  PMID: 25063908 | - Association between several patient and regional demographic, access-related, and provider-related factors, and receipt of any CRC testing procedure (i.e., colonoscopy, FOBT, flexible sigmoidoscopy); stratified by payer type | Colorectal | 27178 | Yes  *(Black vs. White vs. Other)* | No | Medicare, Medicaid | Factors significantly with **increased odds** of CRC testing among **Medicare-only**:   - Turning 50 in 2003-2007 (vs. 2008) - Facility procedure rate >400-600 or >800 per 10,000 county residents (vs. 0) - Female gender (vs. male)   Factors significantly with **decreased odds** of CRC testing among **Medicare-only**:   - African American race (vs. white)   Factors significantly with **increased odds** of CRC testing among **Medicaid-only**:   - Female gender (vs. male) - Turning 50 in 2003-2007 (vs. 2008) - Facility procedure rate >800 per 10,000 county residents (vs. 0) - 2^nd^ quartile of % uninsured (vs. 1^st^ quartile)   Factors significantly with **decreased odds** of CRC testing among **Medicaid-only**:   - African American race (vs. white) - Living >15-20 or >25 mi from facility (vs. ≤5)   Factors significantly with **increased odds** of CRC testing among **dual-enrolled**:   - Female gender (vs. male) - Turning 50 in 2003-2007 (vs. 2008) - 3^rd^ quartile of county % of residents with less than HS education (vs. 1^st^ quartile)   Factors significantly with **decreased odds** of CRC testing among **dual-enrolled**:   - African American race (vs. white) |
| Hsu et al. 2023  PMID: 37222672 | - Association between payer type and cirrhosis etiology with HCC screening | Liver | 46,052 | Yes  (*Black non-Hispanic vs. Hispanic vs.*  *White non-Hispanic vs. Other)* | Yes  *(rural vs. urban by county)* | Medicare, Medicaid, Private | Any HCC screening:   - The 1- and 2-year cumulative incidences 48.5% and 54.7%, respectively - Trended upwards between 2011 and 2016; mostly driven by Medicaid   Screening by ultrasound:   - The 1- and 2-year cumulative incidences: 42.6% and 49.3%, respectively   Any screening; by cirrhosis etiology:   - HBV had the highest 2-year cumulative incidence of HCC surveillance, followed by HCV, NAFLD, ALD, and “Other” cirrhosis etiologies   Proportion of time covered (% of time covered by a surveillance exam, in months, during the follow-up time):   - Mean for all surveillance modalities: 24.9% - Median for all surveillance modalities: 15% - By insurance: for all surveillance modalities, the mean PTC was highest among those with private insurance; For abdominal ultrasound alone, the mean PTC measure was highest among those with Medicaid |

*Race/ethnicity or rurality considered in analysis of the specific topic(s) included in this table; not necessarily reflective of whether race/ethnicity or rurality were considered in the study as a whole.

ALD = alcohol-associated liver disease

FIT = fecal immunochemical test

FOBT = fecal occult blood test

HBV = hepatitis B virus

HCC = hepatocellular carcinoma

HCV = hepatitis C virus

NAFLD = nonalcoholic fatty liver disease

SEHP = state employees health plan

## Theme: Diagnosis/Molecular Testing

| Study | Topic(s) of Study  *(**within theme)* | Cancer Site(s) Studied | Sample Size | Stratified by/Adjusted for Race/Ethnicity?* | Stratified by/Adjusted for Rurality?* | Payer Type | Key Findings |
| --- | --- | --- | --- | --- | --- | --- | --- |
| Reeder-Hayes et al. 2018^  PMID: 29338090 | - Association between race, provider-level factors and GEP testing; stratified by lymph node status | Breast | 11958 | Yes  *(Black vs. Hispanic vs.* *White vs. Other)* | No | Medicare, Private | Overall, 23% of black women and 26.2% of non-Hispanic white women received GEP testing, with **rates rising over time**.  Factors associated with **increased likelihood** of GEP testing, among l**ymph node negative patients:**   - Patient volume ranking of provider (vs. all other providers) in 11^th^-30^th^, 31^st^ – 50^th^, or 51^st^ – 100^th^ (vs. top 10) - 18-49 or 50-59 years old (vs. 60-69 years old) at diagnosis - T1c or T2 tumor classification (vs. T1a-b) - Tumor grade of 2 (vs. 1) - Having any private insurance (vs. Medicare only)   Factors associated with **decreased likelihood** of GEP testing, among **lymph node negative patients:**   - Black (vs. white) race - Provider specialty of general surgery (vs. medical oncology) - 70-79 or ≥80 years old (vs. 60-69 years old) at diagnosis - NCI comorbidity index ≥1 (vs. 0) - Diagnosis in 2005 – 2009 (vs. 2012)   Factors associated with **decreased likelihood** of GEP testing, among **lymph node positive patients:**   - Provider specialty of general surgery or gynecological oncology (vs. medical oncology) - Patient volume ranking of provider (vs. all other providers) in 100^th^-200^th^ (vs. top 10) - ≥80 years old (vs. 60-69 years old) at diagnosis - T2 tumor classification (vs. T1a-b) - Tumor grade of 3 (vs. 1) - Diagnosis in 2005 – 2008 (vs. 2012) |
| Holmes et al. 2012  PMID: 22248516 | - Association between distance to care and diagnosis by race | Prostate | 2251 | Yes  *(Black vs. White)* | Yes  *(**rural vs. urban by county)* | Medicare | **High-risk cancer rate increased with distance** to a urologist and **low-risk cancer rate decreased with distance**.  Magnitude of association of distance was **larger for Black patients** (vs. white patients) |
| Doll et al. 2016 (a)  PMID: 27246688 | - Association between pre-diagnosis Medicaid coverage and stage of diagnosis | Cervical, Ovarian, Uterine, Vaginal,  Gynecologic aggregated | 782 | Yes  *(Black vs. White)* | Yes  *(**metropolitan vs. non-metropolitan by county)* | Medicaid | Among all patients/cancer sites, likelihood of **advanced stage at presentation higher among those without pre-diagnosis Medicaid** coverage (vs. with pre-diagnosis Medicaid coverage). (Not statistically significant for individual cancer sites.) |
| Doll et al. 2015  PMID: 26230631 | - Association between insurance type and stage of diagnosis | Cervical, Ovarian, Uterine, Vaginal,  Gynecologic aggregated | 4552 | Yes  *(Black vs. White)* | Yes  *(**metropolitan vs. non-metropolitan by county)* | Medicare | Among all patients/cancer sites, *no* increased likelihood of advanced-stage disease at the time of diagnosis by insurance type.  Among **uterine cancer patients, dual-enrolled** (vs. Medicare only) patients **more likely to be diagnosed with late-stage** disease. |

*Race/ethnicity or rurality considered in analysis of the specific topic(s) included in this table; not necessarily reflective of whether race/ethnicity or rurality were considered in the study as a whole.

^ Explanation of/results from main variables/associations of interest related to the given topic of study are included in this table; additional variables/associations were also explored in the full study.

GEP = gene expression profiling

# Cancer Care Continuum Stage: Diagnosis

## Theme: Cancer Prevalence Trends

| Study | Topic(s) of Study  *(**within theme)* | Cancer Site(s) Studied | Sample Size | Stratified by/Adjusted for Race/Ethnicity?* | Stratified by/Adjusted for Rurality?* | Payer Type | Key Findings |
| --- | --- | --- | --- | --- | --- | --- | --- |
| Gogate et al. 2021  PMID: 34409255 | - Projecting metastatic breast cancer prevalence through 2030 | Breast | N/A | No | No | N/A | Estimated a **55% increase in the prevalence of metastatic breast cancer** in 2030 relative to 2015. Increase in prevalence expected in women 18-44 and 45-64 yrs old from 2015 to 2030. Initial decrease in prevalence followed by stabilization of prevalence expected for women ≥65 yrs old. |

*Race/ethnicity or rurality considered in analysis of the specific topic(s) included in this table; not necessarily reflective of whether race/ethnicity or rurality were considered in the study as a whole.

# Cancer Care Continuum Stage: Treatment and Survivorship Care

## Theme: Treatment Initiation and Adherence

| Study | Topic(s) of Study  *(**within theme)* | Cancer Site(s) Studied | Sample Size | Stratified by/Adjusted for Race/Ethnicity?* | Stratified by/Adjusted for Rurality?* | Payer Type | Key Findings |
| --- | --- | --- | --- | --- | --- | --- | --- |
| Roberson et al. 2022  PMID: 35286524 | - Trends in type of surgical treatment for early breast cancer (mastectomy vs. breast conserving surgery) - Stratified by race, rurality, age | Breast | 86776 | Yes  *(Black vs. all)* | Yes  *(rural vs. all)* |  | Overall:   - 2003: mastectomy 49.1%; BCS 48.4%; no surgery 2.5% - In 2016, mastectomy 33.7%; BCS 60.8%; no surgery 5.5% - Between 2003 and 2010, no significant change in mastectomy or BSC; between 2010 and 2016, notable decrease in mastectomy and increase in BCS per year. - Black women: No net change in proportion of mastectomy from 2003 to 2011 or BCS from 2003 to 2010; after 2010 and 2011, respectively, the proportion of mastectomy decreased while BCS increased until 2016.   Age groups:   - 18–49 years: mastectomy most common; modest increase in proportion of mastectomy from 2003 to 2016; among Black women BCS remained the more common procedure w/ no net change in proportion - 50–64, and 65+: resembled those seen in the overall population; among Black women mastectomy steadily decreased and BCS procedures increased over the entire time interval   Rural:   - Mastectomy more prevalent procedure type at the start of the - time interval; in 2011 BCS became the more prevalent procedure; BCS proportion remained flat between 2003 and 2010 and then increased from 2010 onward and mastectomy decreased steadily from 2003 to 2011 and then decreased more sharply after 2011 - Black women: observed crossover in most prevalent procedure type happened in 2009; no detected inflections in slope for procedure type, proportion of BCS and mastectomy decreased between 2003 and 2016 with no changes in slope during this time interval |
| Reeder-Hayes et al. 2021  PMID: 33551323 | - Factors associated with receipt of RT - Association between receipt of RT and/or ET and recurrence | Breast | 10204 | Yes  *(Non-Hispanic White vs. Other)* | No | Medicare | Overall, 65.6% of patients received RT  Factors associated with **increased likelihood** of receipt of RT:   - Diagnosis in 2011 (vs. 2007)   Factors associated with **decreased likelihood** of receipt of RT:   - 71-75, 76-80 or >80 yrs old at diagnosis (vs. 66-70) - NCI Combined Comorbidity Index ≥1 (vs. 0) - Residence in Midwest or West US Census Region (vs. South) - T2 tumor (vs. T1) |
| Anderson et al. 2017  PMID: 28408621 | - Factors associated with ET initiation within 1 year of diagnosis | Breast | 2090 | Yes  *(Non-Hispanic Black vs. Non-Hispanic White vs. Other)* | No | Medicare, Medicaid, Private | Factors associated with **increased likelihood** of ET initiation within 1 yr of diagnosis:   - Unknown/not assessed tumor grade (vs. well to moderate) - Saw medical oncology within a yr of diagnosis (vs. not) - HR positive (vs borderline/unknown)   Factors associated with **decreased likelihood** of ET initiation within 1 yr of diagnosis:   - ≥75 yrs old at diagnosis (vs. 45-54) - BCS (w/o RT) or mastectomy (vs. BCS + RT) - HR negative (vs borderline/unknown) |
| Reeder-Hayes et al. 2014  PMID: 24789443 | - Factors associated with adjuvant ET initiation | Breast | 2640 | Yes  *(Black vs. White)* | No | Private | 80% of African American women and 87% of white women initiated RT within 1 yr of diagnosis  Factors associated with **increased likelihood** of ET initiation, among full cohort:   - Stage I at diagnosis (vs. stage III) - Well or moderately differentiated tumor (vs. poorly differentiated)   Factors associated with **decreased likelihood** of ET initiation, among full cohort:   - Black race (vs. white) - BCS w/o RT (vs. BCS + RT) - Receipt of chemotherapy within 1 yr of diagnosis - 2^nd^ (low-mid) quartile % poverty in county of residence (vs. 1^st^ quartile) - *Additional results stratified by previous treatment with CT vs. not.* |
| Wheeler et al. 2014 (b)^  PMID: 25046086 | - Association between distance to care, rurality of residence with receipt of RT | Breast | 1938 | Yes  *(**non-White vs. White)* | Yes  *(**rural vs. urban by county)* | Medicare | Factors associated with **increased likelihood** of receipt of RT:   - Urban residence (vs. rural) - Among rural patients: Living 10-20 mi from nearest RT provider (vs. <10) - Married (vs. not married) - Stage III (vs. stage I)   Factors associated with **decreased likelihood** of receipt of RT:   - 70-74, 75-79, ≥80 years old (vs. 65-69) - State Medicare buy-in vs. no buy-in - Diagnosis in 2004 or 2005 (vs. 2003) - Greater comorbidity index score - For urban patients, living > 20 mi from nearest RT provider (vs. <10) |
| Wheeler et al. 2014 (c)  PMID: 24866922 | - Factors associated with initiation of (guideline concordant) ET | Breast | 222 | Yes  *(Black vs. White vs. Other)* | Yes  *(**urban vs. rural by county)* | Medicaid | Factors associated with **increased likelihood** of guideline-concordant ET initiation:   - Breast and cervical cancer control program participation - Diagnosis in 2004 (vs. 2007) |
| Wheeler et al. 2012  PMID: 22489864 | - Factors associated with receipt and timing of (guideline recommended) RT | Breast | 126 | Yes  *(Black vs. White)* | Yes  *(**urban vs. rural by county)* | Medicaid | Overall, 79% initiated RT within 1 yr of diagnosis  Factors associated with **increased likelihood** of receipt of RT within 180 days of diagnosis:   - Tumor stage I or II (vs. unstaged/missing) - Diagnosis in 2005 (vs. 2003)   Factors associated with **increased likelihood** of receipt of RT within 1 yr of diagnosis:   - Receipt of CT before RT (vs. not)   Factors associated with **decreased likelihood** of receipt of RT within 1 yr of diagnosis:   - Greater NCI comorbidity score   Factors associated with **increased likelihood** of receipt of RT at any time:   - Diagnosis in 2005 (vs. 2003) - Receipt of CT before RT (vs. not)   Factors associated with **decreased likelihood** of receipt of RT at any time:   - Greater NCI comorbidity score   Factors associated with **increased likelihood** of **earlier initiation** of RT among full cohort:   - Stage I tumor (vs. unstaged/missing)   *Additional results stratified by previous treatment with CT vs. not.* |
| Spees et al. 2019 (a)  PMID: 30442384 | - Association between distance to care and receipt of guideline concordant care by rurality | Cervical | 999 | Yes  *(non-White vs. White)* | Yes  *(rural vs. urban by county)* | Medicare, Medicaid, Private | Overall, 62% of patients received guideline concordant care. These patients were generally younger, had private insurance, were less likely to have comorbid conditions, and were more likely to be diagnosed at a later stage.  **Farther distance** (i.e., ≥15 mi) to facility (vs. <5 mi) associated with **lower likelihoo**d of receiving guideline-concordant surgery. Similar trend for only urban residents; no significant association among rural residents.  **Rural residents living 5 to <15 or >15 mi** from facilities (vs. <5 mi) **more likely** to receive brachytherapy. Distance to facility not significantly associated with receipt of brachytherapy overall or among only urban residents.  Distance to nearest facility *not* associated with receipt of CT or EBRT for urban or rural patients. |
| Spees et al. 2019 (b)  PMID: 30733307 | - Factors associated with initiation and completion of treatment; stratified by rurality | Cervical | 999 | Yes  *(non-White vs. White)* | Yes  *(**rural vs. urban by county)* | Medicare, Medicaid, Private | Factors associated with **increased likelihood of treatment initiation** within 6 weeks of diagnosis **among urban patients**:   - Any private insurance (vs. Medicare only)   Factors associated with **decreased likelihood of treatment initiation** within 6 weeks of diagnosis **among urban patients**:   - Living ≥15 mi (vs. <5) from care - >70 yrs old (vs. <60 yrs old) at diagnosis   Factors associated with **decreased likelihood** of treatment **completion** within 8 weeks **among urban patients**:   - >70 yrs old (vs. <60 yrs old) at diagnosis   Factors associated with **increased likelihood of treatment completion** within 8 weeks **among rural patients**:   - Living 5 - <15 or ≥15 mi (vs. <5) to care   Factors associated with **decreased likelihood** of treatment **completion** within 8 weeks among rural patients:   - 60-70 or >70 yrs old (vs. <60 yrs old) at diagnosis |
| Trogdon et al. 2018^  PMID: 29578953 | - Association between provider characteristics and treatment | Colorectal | 7295 | Yes  *(African American vs. White vs. Other)* | Yes  *(rural vs. urban vs. metro)* | Medicare, Medicaid, Private | Factors associated with **increased likelihood of receipt of adjuvant CT**:   - Having MO and surgeon who share 10-20% or >20% (vs. <10%) of their CRC patient volumes with each other |
| Spees et al. 2021  PMID: 34480518 | - Factors associated with OAA initiation - Factors associated with OAA adherence | Kidney | 687 (initiation)  207 (adherence) | Yes  *(non-Hispanic Black vs. non-Hispanic White vs. Other)* | Yes  *(**urban vs. rural by census tract)* | Medicare, Medicaid, Private | Factors associated with i**ncreased likelihood** of OAA **initiation** within one year of metastatic index date:   - Female provider (vs. male) - Providers practicing in urban only areas (vs. urban and rural)   Factors associated with **decreased likelihood** of OAA **initiation** within one yr of metastatic index date:   - ≥80 yrs old (vs. 18-49) - ≥3 comorbid conditions (vs. 0) - Stage I at diagnosis (vs. stage IV) - Frailty above median (vs. below) - Treater specialty of urology/urological surgery, internal medicine, or other (vs. hematology/medical oncology)   Factors associated with **decreased likelihood** of OAA **adherence**:   - Medicare only (vs. private insurance) |
| Wheeler et al. 2021 (a)  PMID: 34138665 | - Factors associated with OAA use | Kidney | 713 | Yes  *(non-Hispanic Black vs. non-Hispanic White vs. Other)* | Yes  *(**rural vs. non-rural by census tract)* | Medicare, Medicaid, Private | Overall, 37% of patients received OAA treatment within 12 mos of diagnosis  Factors associated with **increased likelihood** of OAA use within 1 yr of diagnosis:   - Highest quartile of % poverty in census tract (vs. lower 3 quartiles) - Stage 2 (vs. stage 4) at diagnosis   Factors associated with **decreased likelihood** of OAA use within 1 yr of diagnosis:   - 50-64, 65-69, 70-74, and ≥80 yrs old at diagnosis (vs. 18-49) - 2 or ≥3 comorbid conditions (vs. 0) - Frailty - Stage I or Stage II at diagnosis (vs. Stage IV) |
| Herb et al. 2022 (a)  PMID: 35817447 | - Trends in and factors associated with treatment receipt/type | Lung | 5504 | Yes  *(Hispanic vs. non-Hispanic Black vs. non-Hispanic White vs. Other)* | Yes  *(urban vs. suburban vs. isolated rural by county)* |  | Rural vs. urban   - No significant differences in odds of surgery, RT, or any treatment between rural and urban patients in 2006-2010 or 2011-2015   Rural over time   - Any treatment: no significant change over time - Surgery: lower over time - RT: higher over time   Urban over time   - Any treatment: no significant change over time - Surgery: lower over time - RT: higher over time   Rural only; surgery tertiles   - Highest tertile had shorter distance to care - Highest tertile had lower radiation oncologist density   Rural only; any treatment tertiles   - Highest tertile had shorter distance to care |
| Adamson et al. 2017^  PMID: 28979974 | - Association between patient demographic, access, and clinical factors and delays to surgery | Melanoma | 7629 | Yes  *(non-Hispanic White vs. Other)* | Yes  *(rural vs. non-rural by zip code)* | Medicare, Medicaid, Private | Factors associated with **increased likelihood** of time to surgery >6 weeks:   - Having Medicaid (vs. private) insurance - Other vs. non-Hispanic white race/ethnicity - Cancer stage of II or III (vs. 0) - Tumor location on head and neck or lower extremities (vs. trunk)   Factors associated with **decreased likelihood** of time to surgery >6 weeks:   - Diagnosis by a dermatologist (vs. not) - Surgery by a dermatologist (vs. not) |

*Race/ethnicity or rurality considered in analysis of the specific topic(s) included in this table; not necessarily reflective of whether race/ethnicity or rurality were considered in the study as a whole.

**The study included 1082 cystectomy patients, only 736 of whom had bladder cancer.

^ Explanation of/results from main variables/associations of interest related to the given topic of study are included in this table; additional variables/associations were also explored in the full study.

BCS = breast conserving surgery

CCNC = Community Care of North Carolina

CT = chemotherapy

ET = endocrine therapy

HR = hormone receptor

OAA = oral anticancer agent

RT = radiation therapy

## Theme: Facility/Provider Characteristics

| Study | Topic(s) of Study  *(**within theme)* | Cancer Site(s) Studied | Sample Size | Stratified by/Adjusted for Race/Ethnicity?* | Stratified by/Adjusted for Rurality?* | Payer Type | Key Findings |
| --- | --- | --- | --- | --- | --- | --- | --- |
| Freeman et al. 2016  PMID: 27351768 | - Association between chemotherapy site and mortality | Acute Myeloid Leukemia | 900 | Yes  *(non-Hispanic White vs. Other)* | Yes  *(rural vs. non-rural by zip code)* | Medicare, Medicaid, Private | Factors associated with **increased odds of treatment at a NCICCC**:   - Charlson score ≥1 (vs. 0) - ≥20-mile (vs. <20) distance to treating facility   Factors associated with **decreased odds of treatment at a NCICCC**:   - Household income in the first quartile (vs. fourth) - Enrollment in Medicare only (vs. private insurance or private + Medicare) - Residence in Area L, Charlotte, Eastern, Mountain, or Wake AHEC region (vs. Greensboro) - Undergoing allo-HSCT within 1 yr of diagnosis |
| Trogdon et al. 2018^  PMID: 29578953 | - Association between provider characteristics and likelihood consultation with MO | Colorectal | 7295 | Yes  *(**African American vs. White vs. Other)* | Yes  *(**rural vs. urban vs. metro)* | Medicare, Medicaid, Private | Factors associated with **increased likelihood of consultation with MO**:   - Surgeon shares >40% (vs. <40%) of CRC patients with MO   Factors associated with **decreased likelihood** **of consultation with MO**:   - Treatment by surgeon with 10-30 (vs. <10) CRC patients in past 12 mos |
| Adamson et al. 2017^  PMID: 28979974 | - Association between patient demographic, clinical, and access factors and diagnosis by a dermatologist, surgery by a dermatologist | Melanoma | 7629 | Yes  *(non-Hispanic White vs. Other)* | Yes  *(**rural vs. non-rural by zip code)* | Medicare, Medicaid, Private | 80% of patients diagnosed by dermatologist  Factors associated with **increased likelihood** of **diagnosis** by a dermatologist:   - Age 70-79 or ≥80 years at diagnosis (vs. <50) - Diagnosed in 2008 or 2009 (vs.2004)   Factors associated with **decreased likelihood** of **diagnosis** by a dermatologist:   - Medicare or Medicaid (vs. private) insurance - Other vs. non-Hispanic white race/ethnicity - Rural zip code (vs. non-rural) - ≥1 Charlson comorbidity index - Stage I, II, III, or unknown (vs. stage 0)   Factors associated with **increased likelihood** of **surgery** by a dermatologist:   - Diagnosis by a dermatologist (vs. not) - Diagnosis in 2007, 2008, 2009, 2010, 2011 (vs. 2004)   Factors associated with **decreased likelihood** of **surgery** by a dermatologist:   - Medicaid (vs. private) insurance - Rural zip code (vs. non-rural) - Cancer located on upper or lower extremities (vs. trunk) - Stage I, II, III, or unknown (vs. stage 0) |
| Freeman et al. 2019  PMID: 31487686 | - Factors associated with evaluation at NCICCC | Multiple Myeloma | 1029 | Yes  *(non-Hispanic White vs. Other)* | Yes  *(**rural vs. urban by zip code)* | Medicare, Medicaid, Private | Factors associated with **decreased likelihood** of evaluation at NCICCC:   - Not being married - Age 65-69, 70-74, 75-80, or >80 years (vs. <65) - Medicare or Medicaid (vs. private) insurance - 4^th^ or 3^rd^ quartile of distance to NCICCC (vs. 1^st^ quartile) - 3^rd^ (vs. 4^th^) quartile of % adult population with college degree - 4^th^ or 2^nd^ (vs. 1^st^) quartile of activities of daily living dependency score - Charlson comorbidity index score ≥3 (vs. 0) |
| Doll et al. 2016 (b)  PMID: 27130238 | - Referral patterns, treatment site characteristics, and associated factors | Uterine | 2053 | Yes, just RR outputs  *(non-Hispanic White vs. Other)* | Yes  *(**metro vs. non-metro by county)* | Medicare, Medicaid, Private | Biopsy and surgery referral patterns:   - 40% of patients had initial biopsy at HV centers, 91.7% of whom then had surgery at HV centers - 60% of patients had initial biopsy at LV centers, 67.2% of whom then had surgery at HV centers - Patients with **biopsy at LV center with Medicaid** (vs. private) insurance **less likely to get referred to HV center for treatment**. - Patients with **biopsy at LV center with high-risk** (vs. low-risk) histology **more likely to get referred to HV center for treatment**. - Patients **treated at LV** centers were **more likely to be older, live in non-metro counties, have public insurance, and have higher comorbidity scores.**   Surgery & CT:   - Among patients who received CT and surgery, 65% received both at HV centers (HV-all), 25% received one at a HV and one at a LV center (HV-hybrid), and 11.5% received both at a LV center (HV-none). - **HV-all** patients were **less likely to have public insurance, ≥1 comorbidity and local stage disease** than HV-hybrid or HV-none patients. |
| Stitzenberg et al. 2014  PMID: 24169179 | - Proportion of procedures performed by various surgical specialties by cancer type | Bladder, Breast, Colorectal, Esophageal, Gallbladder, Kidney, Liver, Lung, Melanoma, Ovarian, Pancreatic, Prostate, Small bowel, Stomach, Uterine, Multi-site aggregated | 7759 | No | No | Medicare | Excluding procedures for gynecologic/urologic malignancies, the proportion of procedures performed by general surgeons and surgical oncologists was 48% and 12%, respectively. General surgeons performed more than half of procedures for breast, colorectal, and gallbladder cancer patients.  **Patients treated by general surgeons were more likely to be older, female, minority race/ethnicity, and from areas of high poverty.** |

*Race/ethnicity or rurality considered in analysis of the specific topic(s) included in this table; not necessarily reflective of whether race/ethnicity or rurality were considered in the study as a whole.

^ Explanation of/results from main variables/associations of interest related to the given topic of study are included in this table; additional variables/associations were also explored in the full study.

HSCT = hematopoietic stem cell transplant

HV = high-volume

LV = low-volume

MO = medical oncologist

NCICCC = NCI-designated Comprehensive Cancer Center

## Theme: Recurrence

| Study | Topic(s) of Study  *(**within theme)* | Cancer Site(s) Studied | Sample Size | Stratified by/Adjusted for Race/Ethnicity?* | Stratified by/Adjusted for Rurality?* | Payer Type | Key Findings |
| --- | --- | --- | --- | --- | --- | --- | --- |
| Reeder-Hayes et al. 2021  PMID: 33551323 | - Factors associated with recurrence | Breast | 10204 | Yes  *(non-Hispanic White vs. Other)* | No | Medicare | Factors associated with **increased likelihood** of recurrence:   - 71-75, 76-80, or >80 yrs old (vs. 66-70) - NCI Combined Comorbidity Index ≥1 (vs. 0) - Moderately or poorly differentiated (vs. well-differentiated tumor)   Factors associated with **decreased likelihood** of recurrence:   - RT only and RT+ ET (vs. ET only) - Diagnosed in 2011 (vs. 2007) |

*Race/ethnicity or rurality considered in analysis of the specific topic(s) included in this table; not necessarily reflective of whether race/ethnicity or rurality were considered in the study as a whole.

ET = endocrine therapy

RT = radiation therapy

## Theme: Healthcare Resource Utilization Among Cancer Patients and Survivors

| Study | Topic(s) of Study  *(**within theme)* | Cancer Site(s) Studied | Sample Size | Stratified by/Adjusted for Race/Ethnicity?* | Stratified by/Adjusted for Rurality?* | Payer Type | Key Findings |
| --- | --- | --- | --- | --- | --- | --- | --- |
| Smith et al. 2018^  PMID: 29566978 | - Association between distance to provider and 30-day and 90-day readmission in cystectomy patients | Bladder | 1082** | Yes  *(non-Hispanic White vs. Other)* | No | Medicare, Medicaid, Private | Distance to cystectomy provider *not* significantly associated with likelihood of 30-day or 31-90-day readmission. |
| Kohler et al. 2015^  PMID: 26287506 | - Association between PCMH enrollment and health care utilization | Breast | 758 | Yes  *(Black vs White vs. Other)* | Yes  *(**urban vs. non-urban by county)* | Medicaid | PCMH enrollment *not* significantly associated with inpatient stay or ED visit  PCMH enrollment associated with **increased likelihood** of outpatient visits for reasons other than receipt of cancer treatment. |
| Goyal et al. 2014^  PMID: 25046085 | - Factors associated with health care resource utilization due to CT-related AEs | Breast | 570 | Yes  *(non-Hispanic Black vs. non-Hispanic White vs. Other)* | Yes  *(**urban vs. rural by county)* | Medicaid | **CCNC enrollment associated with decreased likelihood of inpatient admission for a CT-related AE**. (No association between CCNC enrollment and likelihood of outpatient or ED visits.) |
| Check et al. 2022  PMID: 36396793 | - Proportion of patients by cancer site concurrently prescribed opioids and another sedating medication - Factors associated with concurrent prescribing of opioids and other sedating medications in cancer patients | Multi-site aggregated | 14376 | Yes  *(Hispanic vs. non-Hispanic Black vs. non-Hispanic White vs. Other)* | Yes  *(urban vs. rural)* | Medicare, Private | Overall, ~15% of patients had concurrent prescribing of opioids and benzodiazepines or opioids and gabapentinoids; 31% for cervical cancer, 26% for ovarian cancer  Factors associated with **increased** likelihood of concurrent prescribing:   - Age 19-49 or 50-59 (vs. ≥80) - Female - Cervical, head & neck, ovarian, or NCSLC (vs. CRC) - Regional (vs. local) stage - Charlson comorbidity index 1 or ≥2 (vs. 0) - Prior diagnosis or depression, anxiety, substance use disorder, fibromyalgia, or chronic pain - Prior opioid, benzodiazepine, or gabapentinoid use - Receipt of adjuvant chemotherapy or surgery   Factors associated with **decreased** likelihood of concurrent prescribing:   - Age 60-69 or 70-79 (vs. ≥80) - Non-Hispanic Black or Hispanic race/ethnicity (vs. non-Hispanic White) - Medicare only (vs. any private) - Melanoma (vs. CRC)   *Additional data presented for factors associated with specific drug combinations (opioid/benzodiazepine, opioid/gabapentinoid)* |
| Check et al. 2021  PMID: 33881543 | - Patterns of opioid use during treatment and in early survivorship phase - Factors associated with chronic opioid use in cancer survivors | Multi-site aggregated | 38336 | Yes  *(non-Hispanic Black vs. non-Hispanic White vs. Hispanic or Other)* | Yes  *(rural vs. urban)* | Medicare, Medicaid, Private | During cancer treatment: 51.5% of cancer survivors used no opioids, 46.0% used opioids intermittently, and 2% used opioids chronically  In early survivorship period, **patients who were opioid naïve prior to cancer diagnosis** (79.9%) **and then used opioids intermittently during treatment** (39.1%) **largely returned to non-use** in early survivorship (64.0%); 33.9% continued to use opioids intermittently in early survivorship, and 2.1% progressed to chronic use  In early survivorship period, **patients who were intermittent opioid users prior to cancer** (16.9%) **and during active cancer treatment** (78.7%) **largely remained intermittent users** in early survivorship (54.9%); 5.9% progressed to chronic use  Factors associated with **increased likelihood** of chronic opioid use in early survivorship period:   - Age 50-59 years (vs. 60-69) - 3^rd^ (medium-high) quartile (vs. 4^th^ (high) quartile) of census tract-level % with at least a HS diploma - Medicaid or Medicare (vs. private) insurance - NSCLC diagnosis (vs. CRC) - Charlson comorbidity index ≥2 (vs. 0) - Prior depression diagnosis (vs. not) - Prior substance use disorder diagnosis (vs. not) - Prior diagnosis of fibromyalgia or chronic pain (vs. not) - Prior intermittent opioid use (vs. not) - Long-term opioid use -1 to 12 mo post-diagnosis (vs. non-long-term use)   Factors associated with **decreased likelihood** of chronic opioid use in early survivorship period:   - Age 70-79 years (vs. 60-69) |
| Spees et al. 2020  PMID: 32780539 | - Association between cancer diagnosis and chronic medication adherence, hospitalization among patients with chronic conditions; stratified by PCMH use | Multi-site aggregated | 688  (w/ diabetes)  1445  (w/ hyper-tension)  955  (w/ hyper-lipidemia) | Yes  *(non-Hispanic Black vs. non-Hispanic White vs. Other)* | No | Medicare, Medicaid | Chronic medication adherence:   - Both pre- and post-diagnosis medication **adherence were highest** among patients with **hypertension** and were **lowest** among patients with **hyperlipidemia** - Among patients with hyperlipidemia, statin **adherence decreased more between pre-diagnosis and the first year post-diagnosis in cancer patients** (vs. those without cancer). - Changes in medication adherence over time between patients with and without cancer were *not* significantly different across PCMH status for any of the chronic condition cohorts   Inpatient hospitalization:   - Across chronic condition cohorts, **hospitalization occurred more often for cancer patients pre-diagnosis** than for non-cancer patients - Across chronic condition cohorts, **increases in the likelihood of hospitalization in first 6 mos post-diagnosis (vs. pre-diagnosis) was larger for cancer patients** compared to non-cancer patients. - Across chronic condition cohorts, **cancer patients’ likelihood of hospitalizations declined in mos 7-12 post-diagnosis** relative to pre-diagnosis. - PCMH and non-PCMH cancer patients had increases in likelihood of hospitalization in the first 6 mos post-diagnosis (vs. pre-diagnosis) relative to non-cancer patients. - In the hypertension cohort, **increases in inpatient hospitalization rates were larger for PCMH cancer patients than for non-PCMH cancer** patients (vs. non-cancer patients).   ED visits:   - Declines in the **likelihood of an ED visit** in the first 6 mos or mos 7-12 post-diagnosis (vs. pre-diagnosis) were s**imilar between cancer and non-cancer patients** with one exception: for cancers patients with hypertension, the likelihood of ED visits decreased in mos 7-12 post-diagnosis (vs. non-cancer patients with hypertension). - PCMH and non-PCMH cancer patients experienced *no* statistically significant changes in ED, both between the first 6 mos and mos 7-12 post-diagnosis (vs. pre-diagnosis) |
| Williams et al. 2019  PMID: 30870086 | - Association between geriatric assessment-identified impairments and hospitalization or long-term care use in cancer survivors | Multi-site aggregated | 125 | No | No | Medicare | Factors associated with **increased likelihood** of hospitalization   - Any IADL impairment (vs. none) - Karnofsky performance status <70 (vs. ≥70) - Prolonged TUG (vs. normal) - Limited in climbing stairs (vs. not) - Limited in walking one block (vs. not) - Weight loss >5% (vs. not) - Prefrail/frail (vs. robust)   Factors associated with **increased likelihood** of long-term care use:   - Any IADL impairment (vs. none) - ≥1 falls (vs. 0) - Karnofsky performance status <70 (vs. ≥70) - Prolonged TUG (vs. normal) - Limited in climbing stairs (vs. not) - Limited in walking one block (vs. not) - Weight loss >5% (vs. not) - Prefrail/frail (vs. robust)   Factors associated with **decreased likelihood** of long-term care use:   - Taking >9 (vs. ≤9) medications - Has anxiety (vs. not) |
| Pergolotti et al. 2014  PMID: 25184473 | - Factors associated with occupational therapy use | Multi-site, aggregated | 27131 | Yes  *(African American vs. White vs. Other)* | Yes  *(Larger urban vs. metropolitan vs. rural by county)* | Medicare, Medicaid, private | Overall, 32% patients used OT within the first 2 yrs of cancer diagnosis  Factors associated with **increased likelihood** of use of OT:   - Age by 5 yr increments - Female sex (vs. male) - Years of education - White (vs. non-African American other) race - Dual eligibility - Previous use of OT - Breast cancer, CRC, or melanoma (vs. prostate cancer) - Breast cancer, CRC, prostate cancer, or melanoma (vs. lung cancer) - Stage I, II, or III (vs. unknown stage and vs. Stage 0) - Stage 0, I, II, and III (vs. Stage IV) - CCI 1, 2, 3 or ≥4 (vs. 0) - CCI 3 or ≥4 (vs. 1 and vs. 2) |

*Race/ethnicity or rurality considered in analysis of the specific topic(s) included in this table; not necessarily reflective of whether race/ethnicity or rurality were considered in the study as a whole.

**The study included 1082 cystectomy patients, only 736 of whom had bladder cancer.

^ Explanation of/results from main variables/associations of interest related to the given topic of study are included in this table; additional variables/associations were also explored in the full study.

CCI = Charlson Comorbidity Index

CCNC = Community Care of North Carolina

CRC = colorectal cancer

CT = chemotherapy

HS = high school

IADL = instrumental activities of daily living

OT = occupational therapy

PCMH = patient-centered medical home

SNF = skilled nursing facility

TUG = timed up and go

## Theme: Follow-Up Care

| Study | Topic(s) of Study  *(**within theme)* | Cancer Site(s) Studied | Sample Size | Stratified by/Adjusted for Race/Ethnicity?* | Stratified by/Adjusted for Rurality?* | Payer Type | Key Findings |
| --- | --- | --- | --- | --- | --- | --- | --- |
| Roughton et al. 2016  PMID: 27465180 | - Factors associated with receipt and timeliness of post-mastectomy breast reconstruction | Breast | 5381 | Yes  *(non-Hispanic White vs. Other)* | Yes  *(rural vs. non-rural by county)* | Medicare, Medicaid, Private | Overall, 21% of women underwent breast reconstruction.  Factors associated with **increased likelihood** of receipt of breast reconstruction:   - Diagnosis in 2004 or 2006 (vs. 2003)   Factors associated with **decreased likelihood** of receipt of breast reconstruction:   - Increased age at diagnosis - Stage II/III (vs. 0/I) - Living 10-20 or ≥20 mi from surgeon (vs. <10 mi) - Any Medicaid or private + Medicare or Medicare (vs. private only) - 1^st^ or 2^nd^ quartile of census tract-level median income (vs. 4^th^ quartile) - Race other than non-Hispanic white (vs. non-Hispanic white) - Receipt of RT (vs. not) - Living in a rural county (vs. not)   Factors associated with **increased likelihood** of **delayed** receipt of breast reconstruction:   - Receipt of chemotherapy (vs. not) - Receipt of RT (vs. not) - Living in a rural county (vs. not) - Autologous reconstruction (vs. implant-based) |
| Wheeler et al. 2013 ^  PMID: 23673393 | - Association between enrollment in CCNC and receipt of guideline concordant follow-up care | Breast | 840 | Yes  *(Black vs. Hispanic vs. White vs. Other vs. Multiple races/ethnicities)* | Yes  *(urban vs. rural by county)* | Medicaid | Overall ~half of women were enrolled in CCNC at some point  **Increased duration of CCNC enrollment** associated with **increased likelihood of receipt of follow-up mammogram** w/in 15 mos post-diagnosis or within 15 mos of receipt of RT  No significant differences in the likelihood of receipt of 2 physical exams by time enrolled in CCNC |
| Trogdon et al. 2018^  PMID: 29578953 | - Association between provider characteristics and treatment follow up | Colorectal | 7295 | Yes  *(African American vs. White vs. Other)* | Yes  *(rural vs. urban vs. metro)* | Medicare, Medicaid, Private | Factors associated with **increased likelihood of surveillance colonoscopy** within **12** mos:   - Treatment by surgeon with 10-30 (vs. <10) CRC patients in past 12 mos - Mean of surgeon % CRC patient volume shared with MO and MO % CRC patient volume shared with surgeon of >20% (vs. <10%)   Factors associated with **decreased likelihood of surveillance colonoscopy** within **12** mos:   - Surgeon shares >40% (vs. <40%) of CRC patients with MO   Factors associated with **increased likelihood of surveillance colonoscopy** within **18** mos:   - Treatment by surgeon with 10-30 (vs. <10) CRC patients in past 12 mos - Mean of surgeon % CRC patient volume shared with MO and MO % CRC patient volume shared with surgeon of >20% (vs. <10%) |

*Race/ethnicity or rurality considered in analysis of the specific topic(s) included in this table; not necessarily reflective of whether race/ethnicity or rurality were considered in the study as a whole.

^ Explanation of/results from main variables/associations of interest related to the given topic of study are included in this table; additional variables/associations were also explored in the full study.

CCNC = Community Care of North Carolina

RT = radiation therapy

# Cancer Care Continuum Stage: Patient and Population Outcomes

## Theme: Mortality and Survival

| Study | Topic(s) of Study  *(**within theme)* | Cancer Site(s) Studied | Sample Size | Stratified by/Adjusted for Race/Ethnicity?* | Stratified by/Adjusted for Rurality?* | Payer Type | Key Findings |
| --- | --- | --- | --- | --- | --- | --- | --- |
| Freeman et al. 2016  PMID: 27351768 | - Association between demographic factors, patient characteristics, treatment characteristics, distance to treatment care and 1-year mortality | Acute Myeloid Leukemia | 900 | Yes  *(non-Hispanic White vs. Other)* | Yes  *(rural vs. non-rural by zip code)* | Medicare, Medicaid, Private | Factors associated with **increased risk of mortality**:   - Age 60-69, 70-79 and ≥80 yrs (vs.19-40) - Residence in Charlotte, Wake, Area L, of Eastern AHEC region (vs. Greensboro) - Charlson Comorbidity Index ≥1 (vs. 0) - Receipt of only outpatient or no chemotherapy (vs. inpatient) - Not undergoing allo-HSCT within 1 yr of diagnosis   *Additional results stratified by treatment with inpatient CT vs. not.* |
| Smith et al. 2018  PMID: 29566978 | - Association between patient demographic, clinical, and access-related factors and survival in cystectomy patients | Bladder | 1082** | Yes  *(non-Hispanic White vs. Other)* | No | Medicare, Medicaid, Private | Factors associated with **decreased** likelihood of long-term survival   - Readmission 31-90 days post-cystectomy discharge - Ta-Tis-TX or T3-T4 pathalogical stage (vs. T0-T2) - Charlson Comorbidity Index of 1 or ≥2 (vs. 0) - Any of pulmonary, cardiac, acute renal failure, venous thromboembolism, gastrointestinal, sepsis, wound, iatrogenic injury, or hemorrhage as a post-cystectomy complication |
| Trogdon et al. 2018^  PMID: 29578953 | - Association between provider characteristics and treatment, outcomes | Colorectal | 7295 | Yes  *(African American vs. White vs. Other)* | Yes  *(rural vs. urban vs. metro)* | Medicare, Medicaid, Private | Surgeon CRC patient volume in prior 12 mo, % of surgeon CRC patient volume shared with MO, MO CRC patient volume in prior 12 mo, % MO CRC patient volume shared with surgeon, and mean surgeon and MO % shared volume are ***not* significantly associated with 5-yr overall survival**. |
| Spees et al. 2022  PMID: 35595633 | - Patient and provider factors associated with survival | Kidney | 207 | Yes  *(non-Hispanic White vs. other)* | Yes  *(urban vs. rural by census tract)* | Medicare, Medicaid, Private | Factors associated with **increased risk of mortality**:   - Having only Medicare (vs. any private) insurance - De novo vs. recurrent metastatic diagnosis - Frailty (Faurot algorithm) |
| Adamson et al. 2021  PMID: 33098016 | - Association between timeliness of surgical excision and overall survival | Melanoma | 6477 | Yes  *(non-Hispanic White vs. other)* | Yes  *(urban vs. rural by zip code)* | Medicare, Medicaid, Private | **Survival probabilities were lower with increasing surgical delay tim**e throughout the course of follow-up (up to 5 yrs)  Absolute **differences in survival probabilities** for those undergoing excision 90-365 days (vs. < 6 weeks) were significantly different and **increased over time**. (Similar, but not statistically significant, trend for excision 6 weeks – 90 days vs. <6 weeks.)  *Additional results stratified by stage of disease.* |
| Freeman et al. 2019  PMID: 31487686 | - Associations between evaluation at NCICCC and overall survival | Multiple Myeloma | 1029 | Yes  *(non-Hispanic White vs. other)* | Yes  *(rural vs. urban by zip code)* | Medicare, Medicaid, Private | Those **not evaluated at an NCICCC** (vs. those who were) and those **treated by a HV or LV community oncologis**t (vs. NCICCC MM specialist) had a **higher risk of mortality**. The higher risk of mortality persisted regardless of if the community oncologist had a history of patient sharing with NCICCC MM specialists. |
| Doll et al. 2016 (b)  PMID: 27130238 | - Association between referral patterns, treatment site characteristics, and outcomes | Uterine | 2053 | Yes, just RR outputs  *(non-Hispanic White vs. other)* | Yes  *(metro vs. non-metro by county)* | Medicare, Medicaid, Private | In patients who received CT and surgery, those who received both at HV centers had higher survival probability than those who received just one or neither at a HV center. |
| Doll et al. 2016 (a)  PMID: 27246688 | - Association between pre-diagnosis Medicaid coverage and mortality | Cervical, Ovarian, Uterine, Vaginal, Gynecologic aggregated | 782 | Yes  *(Black vs. White)* | Yes  *(**metropolitan vs. non-metropolitan by county)* | Medicaid | **Increased risk of mortality** among those with **advanced stage** of disease (vs. local)  *No* statistically significant association between pre-diagnosis Medicaid and mortality. |
| Doll et al. 2015  PMID: 26230631 | - Association between insurance type and mortality | Cervical, Ovarian, Uterine, Vaginal,  Gynecologic aggregated | 4552 | Yes  *(Black vs. White)* | Yes  *(**metropolitan vs. non-metropolitan by county)* | Medicare | **Dual-enrolled** (Medicare + Medicaid) (vs. Medicare-only patients) are at **increased risk of mortality among** patients diagnosed with **uterine, ovarian, and vulvar/vaginal**. |

*Race/ethnicity or rurality considered in analysis of the specific topic(s) included in this table; not necessarily reflective of whether race/ethnicity or rurality were considered in the study as a whole.

^ Explanation of/results from main variables/associations of interest related to the given topic of study are included in this table; additional variables/associations were also explored in the full study.

**The study included 1082 cystectomy patients, only 736 of whom had bladder cancer.

CRC = colorectal cancer

CT = chemotherapy

HV = high volume

LV = low volume

MO = medical oncologist

NCICCC = NCI-designated Comprehensive Cancer Center

# Cancer Care Continuum Stage: Cross-Cutting Themes

## Theme: Distance to Care

| Study | Topic(s) of Study  *(**within theme)* | Cancer Site(s) Studied | Sample Size | Stratified by/Adjusted for Race/Ethnicity?* | Stratified by/Adjusted for Rurality?* | Payer Type | Key Findings |
| --- | --- | --- | --- | --- | --- | --- | --- |
| Stitzenberg et al. 2014  PMID: 24169179 | - Distance to care by surgeon type (general vs. specialist) | Breast, Colorectal, Esophageal, Gallbladder, Liver, Lung, Melanoma, Pancreatic, Stomach | 7759 | No | No | Medicare | For each tumor type, **distance to care was longer for patients seeing specialists** vs. general surgeons. |

*Race/ethnicity or rurality considered in analysis of the specific topic(s) included in this table; not necessarily reflective of whether race/ethnicity or rurality were considered in the study as a whole.

## Theme: Costs of Care

| Study | Topic(s) of Study  *(**within theme)* | Cancer Site(s) Studied | Sample Size | Stratified by/Adjusted for Race/Ethnicity?* | Stratified by/Adjusted for Rurality?* | Payer Type | Key Findings |
| --- | --- | --- | --- | --- | --- | --- | --- |
| Gogate et al. 2021  PMID: 34409255 | - Projecting metastatic breast cancer costs through 2030 | Breast | N/A | No | No | N/A | **Estimated total costs** (medical and productivity) of mBC across all age groups, and phases of care was $63.4 billion in 2015 and $152.4 billion in 2030, an **increase of 140%**.  2030 estimated costs by age group were $75.3 billion for women 45 to 64 yrs old, $42.4 billion for women 18 to 44 yrs old, and $34.7 billion for women 65 yrs old or older.  Estimated 2030 productivity costs were similar to medical costs for women aged 18 to 44 yrs, ~one-quarter lower for women aged 45 to 64 yrs, and ~one-half lower for women aged 65 yrs or older. |
| Wheeler et al. 2021 (b)  PMID: 33558179 | - Cost-effectiveness of ET, RT, and ET+RT | Breast | 10000 | No | No | Medicare | **Total costs per person and QALYs per person were similar for ET only, RT only, and ET + RT groups** (cost range: $216,205-$216,912; QALY range: 7.369-7.395). (Differences in costs and QALYs were <1% of totals.)  ICER:   - RT only (vs. ET only): $10,826/QALY gained - ET + RT (vs. ET only): $26,834/QALY gained - ET + RT (vs. RT only): $60,691/QALY gained |
| Trogdon et al. 2020  PMID: 32346820 | - Average medical costs by age and treatment phase | Breast | 26577 | No | No | Medicare, Medicaid, Private | Expected monthly **costs for women with mBC were significantly higher than for earlier stage** breast cancer **and non-cancer** controls **for all age groups** (18-44, 45-64, 65+) **and treatment phases** (initial, continuing, terminal) *except* during the initial treatment phase among women with stage 3 breast cancer at diagnosis.  Within each age group, the **incremental average monthly costs of mBC** during the initial treatment phase **decreased as comparator stage increased**.  Within each treatment phase, the incremental average monthly costs of mBC were *not* statistically significantly different across age groups. |
| Allaire et al. 2017  PMID: 28432514 | - Cost of care in the first 18 months of treatment (and excess costs vs. no cancer) by stage of diagnosis, service type | Breast | 1106 (w/ cancer) | No | No | Private | Private insurance costs were significantly higher among women aged 18-44 with breast cancer (vs. without breast cancer) for all services (physician office, inpatient hospital, outpatient hospital, prescription drugs) at all points in time (6, 12, 18 mos). Hospital outpatient and physician services costs were the largest drivers at all time periods.  The excess costs at 6 mos (vs. no cancer) were $65,189, at 12 mos were $97,486 and at 18 mos were $108,857.  Among women aged 45-64 with breast cancer, excess cost (vs. without breast cancer) at 12 mos were $75,737. Hospital outpatient and physician services costs were the largest drivers.  Excess cost at 12 mos were significantly higher among women aged 18-44 than 45-64. |
| Trogdon et al. 2017  PMID: 28702893 | - Excess costs among cancer patients (vs. no cancer) in the first 6 months post-diagnosis by age, race, and stage of disease | Breast | 456 (w/ cancer) | Yes  *(Black vs. White)* | No | Medicaid | **Costs** were significantly **higher among women with breast cancer than those without** breast cancer for all stages (localized, regional, distant). The excess costs, relative to no cancer, at 6 mos after diagnosis were $37,114 for women 18-44 yrs old and $28,026 for women 45-64 yrs old.  **Excess costs at 6 mos increased with cancer stage** for localized, regional, and distant cancer stages at 6 mos, respectively, for women aged 18-44 and 45-64. Estimated excess cost in 6 mos post-diagnosis was significantly higher to treat localized and regional (but not distant) cancer among women aged 18-44 (vs. 45-64).  No statistically significant differences in excess costs of breast cancer by race for women aged 18-44 or 45-64. |
| Kohler et al. 2015  PMID: 26287506 | - Association between PCMH enrollment and health care costs | Breast | 758 | Yes  *(Black vs. White vs. Other)* | Yes  *(**urban vs. non-urban by county)* | Medicaid | **PCMH enrollment** corresponded to an **increase in average monthly expenditures** of $429 per mo **in the first 15 mos**  PCMH effect on expenditures was not statistically significant at 24 or 36 mos after diagnosis; the magnitude of the difference in monthly expenditures between PCMH-enrolled and non-PCMH-enrolled patients trended downward over time |
| Mitchell et al. 2020  PMID: 31611329 | - Association between practice setting (NCICCC vs. non-NCICCC) and use of high-cost treatment options | Colorectal, Head & Neck, Lung, Multi-site aggregated | 800 | Yes  *(**White vs. non-White)* | No | Medicare, Private | **Privately-insured** patients **not treated at NCICCC more likely to receive high-cost treatments** than privately-insured patients treated at NCICCC**.**  *No* statistically significant differences between NCICCC and non-NCICCC in high-cost treatment use overall or by individual cancer type. |
| Spees et al. 2020  PMID: 32780539 | - Association between cancer diagnosis and cost of care among patients with chronic conditions; stratified by PCMH use | Multi-site aggregated | 688  (w/ diabetes)  1445  (w/ hyper-tension)  955  (w/ hyper-lipidemia) | Yes  *(non-Hispanic Black vs. non-Hispanic white vs. Other)* | No | Medicare, Medicaid | Across all chronic conditions (diabetes, hypertension, hyperlipidemia), **patients with cancer (vs. without cancer) were noted to experience significant increases in their medical expenditures** in the first year post-diagnosis.  **Increases in medical spending were smaller for PCMH patient**s with cancer compared with non-PCMH patients with cancer (vs. with patients without cancer), particularly within the diabetes cohort. |

*Race/ethnicity or rurality considered in analysis of the specific topic(s) included in this table; not necessarily reflective of whether race/ethnicity or rurality were considered in the study as a whole.

^ Explanation of/results from main variables/associations of interest related to the given topic of study are included in this table; additional variables/associations were also explored in the full study.

mBC = metastatic breast cancer

ET = endocrine therapy

ICER = incremental cost-effectiveness ratio

NCICCC = NCI-designated Comprehensive Cancer Center

PCMH = patient-centered medical home

QALY = quality-adjusted life-year

RT = radiation therapy

# Non-Cancer Care Continuum Themes

## Theme: Data Validity and Utility

| Study | Topic(s) of Study  *(**within theme)* | Cancer Site(s) Studied | Sample Size | Includes Race/Ethnicity Data?* | Includes Rurality Data?* | Payer Type | Key Findings/Conclusions |
| --- | --- | --- | --- | --- | --- | --- | --- |
| Roberson et al. 2022  PMID: 34783925 | - Assessing validity of cancer registry data about receipt of surgery relative to insurance claims data | Breast | 26819 | Yes  *(Black vs. non-Black)* | Yes  *(**urban vs. rural by census tract)* | Medicare, Medicaid, Private, Other Federal, Uninsured | Compared to the overall breast cancer population in NC, the **population that linked to claims was comparably Black and rural**, had an **older age** distribution, and an **overrepresentation of Medicare enrollees**  Overall **sensitivity** (probability of having surgery listed in the registry given an insurance claim for surgery) **was 97.9% and PPV** (probability of having an insurance claim for surgery given surgery was listed in the registry) **was 93.2%**. High sensitivity and PPV were also seen in patients in rural areas and Black patients. **Sensitivity and PPV were lower for Medicaid patients** than patients with Medicare or private insurance.  Among those with both claims and registry, there was a **high agreement between the surgery type** recorded in the cancer registry and the type in the insurance claims  Overall specificity was 47.6%. Specificity was lowest among those with private insurance (14.6%). |
| Herb et al. 2022 (b)  PMID: 36030606 | - Evaluate which SES measure best improves model ability to predict receipt of guideline concordant care and overall survival | Colorectal | 9574 | Yes  *(Asian vs. Black vs. White vs. Other)* | No | Private, Public | Guideline concordant care:   - Social Deprivation Index, Social Vulnerability Index, and Area Deprivation Index models all had significantly improved model fit on likelihood ratio testing compared to the baseline model; Distressed Community Index model did not - Models for all four indices had same area under the curve - Social Deprivation Index and Social Vulnerability Index were associated with likelihood of guideline concordant treatment (increased deprivation associated with lower treatment likelihood); Area Deprivation Index and Distressed Community Index not associated with likelihood of guideline concordant treatment   Overall Survival:   - Social Deprivation Index, Social Vulnerability Index, Area Deprivation Index, and Distressed Community Index models all had significantly improved model fit compared to base model on log likelihood testing - Models for all four indices had same area under the curve - All four indices associated with overall survival (increased deprivation associated with worse survival) |
| Trogdon et al. 2019  PMID:30945065 | - Quantifying measures of care coordination and patient sharing among providers; comparing outcomes from different payer databases | Colorectal | 33164 *(patient sharing relation-ships)* | No | No | Medicare, Private | Medicare claims missed 14.6% of and private insurance claims missed 54.9% of all shared patient relationships.  Patients in the **private insurance** (vs. Medicare) database were **more likely to be shared among at least two different specialties**.  **Medicare providers shared patients with a greater number of other providers** than providers in the private insurance database, but **providers in the private insurance database shared a higher fraction of their patients with other providers**  Clustering coefficients for providers, weighted betweenness, and eigenvector centrality **varied greatly across payers.**  The **relative position of providers in the distribution of patient volumes differed across payers** |
| Anderson et al. 2020  PMID: 31811983 | - Assessing validity of cancer registry treatment data relative to insurance claims data | Breast, Cervical/ Uterine, Hodgkin Lymphoma, Non-Hodgkin Lymphoma, Ovarian, Multi-site, aggregated | 2342 | No | No | Medicaid, Private | Chemotherapy:   - **For all cancer types, the sensitivity was 86.4%**. It was lowest for cervical/uterine cancer and highest for Hodgkin lymphoma - **For all cancer types, PPV was 81.9%**. It was lowest for cervical/uterine and highest for breast cancer. - The kappa statistic (48.8%) and percent agreement (78.0%) indicated weak to moderate overall concordance between CT in claims data and registry data. - **Sensitivity and PPV lower among women with localized** disease (vs. regional or distant stage disease). - Date of initiation in the registry exactly matched the date of the first CT claim for 37%; dates differed by more than 30 days for 37%.   Radiation:   - **For all cancer types, sensitivity for radiation receipt was 74.4%**. It was lowest for Hodgkin lymphoma and highest for cervical/uterine. - **PPV was 83.0% for all cancer types** combined. It was lowest for thyroid and highest for Hodgkin lymphoma. - Kappa and percent agreement were 59.3% and 79.7%, respectively. - **Sensitivity was slightly higher and the PPV was slightly lower, among women with private insurance** **only** (vs. any Medicaid). - Sensitivity, kappa, and percent agreement were all highest among women with localized stage disease; PPV varied little by stage. - Date of initiation in the registry exactly matched the date of the first radiation claim for 28%; dates differed by more than 30 days for 6%   Hormone Therapy (breast only):   - **Sensitivity and PPV for hormone therapy data were 67.0% and 70.1%, respectively**. Kappa (45.7%) and percent agreement (74%) suggested minimal to weak agreement between the registry and the claims data. - Date of initiation in the registry exactly matched the date of the first radiation claim for 31%; dates differed by more than 30 days for 23% |
| Nichols et al. 2021  PMID: 33619021 | - Assemble a cohort of adolescent and young adult women cancer survivors | Multi-site, aggregated | 11072 | Yes  *(Race: African American vs. Asian vs. White vs. Other vs. unknown; Ethnicity: Hispanic vs. non-Hispanic)* | Yes  *(**urban vs. rural by county)* | Private | **Passive data collection** through linkage **reduces participant burden** and **prevents systematic cohort attrition or potential selection biases** that can occur with active participation requirements. |
| Lund et al. 2017  PMID: 28408619 | - Using data linkage to compile a geriatric oncology data resource for researchers | Multi-site, aggregated | 369 | Yes  *(**White vs. non-White)* | No | Medicare | Shows **potential for data linkage to improve the characterization of health status** among older adults with cancer and the **possibility to conduct passive follow‐up** for outcomes of interest over time. |
| Carpenter et al. 2012  PMID:22761398 | - Development of a surveillance system for clinical trial enrollment and targeting | Multi-site, aggregated | 479123 (total)  11362  (in trials) | Yes  *(White vs. minority)* | Not explicitly, though AHEC region included | Insured vs. Not insured | The NCI treatment trial enrollment rate was 2.39% for whites and 2.20% for minorities from 1996-2007, and 2.88% and 2.47%, respectively, for 2005-2007.  **Minority men had the lowest enrollment rate** at 1.33%, while the enrollment rate of white women was highest at 3.21%.  Enrollment rates among all races and genders except among minority men were higher in 2005-2007 relative to 1996-2007.  **Counties with NCI CCOP-affiliated practices and medical schools typically had greater clinical trial enrollment**  Counties with a CCOP or a medical school tended to have a greater proportion of minorities, and in these counties, there was a comparable proportion of minorities in the incident cancer population and the clinical trial population |

*Race/ethnicity or rurality considered in analysis of the specific topic(s) included in this table; not necessarily reflective of whether race/ethnicity or rurality were considered in the study as a whole.

CCOP = Community Clinical Oncology Program

CT = chemotherapy

PPV = positive predictive value
